# Intravascular imaging-guided versus angiography-guided percutaneous coronary intervention: a systematic review and bayesian network meta-analysis of randomized controlled trials

**DOI:** 10.1186/s12872-024-04105-5

**Published:** 2024-09-11

**Authors:** Ahmed Mazen Amin, Yehya Khlidj, Mohamed Abuelazm, Ahmed Sayed, Ubaid Khan, Mariam Mahmoud Elewidi, Mohammad Tanashat, Hesham Elharti, Mohamed Hatem Ellabban, Abdullah K. Alassiri, Mohamad Alsaed, Basel Abdelazeem, Akram Kawsara

**Affiliations:** 1 Faculty of Medicine, Mansoura University, Mansoura, Egypt; 2 Faculty of Medicine, Algiers University, Algiers, Algeria; 3 Faculty of Medicine, Tanta University, Tanta, Egypt; 4 Faculty of Medicine, Ain Shams University, Cairo, Egypt; 5 Division of Cardiology, University of Maryland School of Medicine, Baltimore, Maryland USA; 6 Faculty of Medicine, Yarmouk University, Irbid, Jordan; 7 Faculty of Medicine, Al-Azhar University, Cairo, Egypt; 8 Faculty of Medicine, King Abdulaziz University, Jeddah, Saudi Arabia; 9 Department of Medicine, West Virginia University, Morgantown, WV USA; 10 Department of Cardiology, West Virginia University, Morgantown, WV USA; 11 Houston Methodist DeBakey Heart & Vascular Center, Houston, Texas USA

**Keywords:** Intravascular ultrasound, Optical coherence tomography, Angiography, Coronary artery disease, Percutaneous coronary intervention

## Abstract

**Background:**

Percutaneous coronary intervention (PCI) has become one of the most commonly performed interventional life-saving procedures worldwide. Intravascular Imaging (intravascular ultrasound (IVUS) and optical coherence tomography (OCT)) have initially evolved to guide PCI compared with angiography. However, this technology is not universally employed in all PCI procedures, and there is ongoing controversy regarding its additional benefits to patient outcomes. We aim to estimate the efficacy and safety of imaging modalities during PCI, allowing pre-, per, and post-intervention assessment of coronary vascularization.

**Methods:**

A systematic review and Bayesian network meta-analysis of randomized controlled trials (RCTs), which were retrieved from PubMed, WOS, SCOPUS, EMBASE, and CENTRAL through September 2023. We used R, version 4.2.0. Effect sizes will be presented as odds ratios with accompanying 95% credible intervals. PROSPERO ID: CRD42024507821.

**Results:**

Our study, encompassing 36 RCTs with a total of 17,572 patients, revelead that compared to conventional angiography, IVUS significantly reduced the risk of major adverse cardiovascular events (MACE) (OR: 0.71 [95% CrI: 0.56 to 0.87]) but not OCT (OR: 0.91 [95% CrI: 0.62 to 1.39]), IVUS and OCT significantly reduced the risk of cardiac death (OR: 0.50 [95% CrI: 0.33 to 0.76]) and (OR: 0.55 [95% CrI: 0.31 to 0.98]), respectively, IVUS significantly reduced the risk of target vessel-related revascularization (OR: 0.60 [95% CrI: 0.48 to 0.75]) but not OCT (OR: 0.86 [95% CrI: 0.60 to 1.19]), IVUS and OCT significantly reduced the risk of stent thrombosis (OR: 0.50 [95% CrI: 0.28 to 0.92]) and (OR: 0.48 [95% CrI: 0.22 to 0.98]), respectively, IVUS significantly reduced the risk of re-stenosis (OR: 0.65 [95% CrI: 0.46 to 0.88]) but not OCT (OR: 0.55 [95% CrI: 0.15 to 1.99]), neither IVUS (OR: 0.97 [95% CrI: 0.71 to 1.38]) nor OCT (OR: 0.75 [95% CrI: 0.49 to 1.22]) were associated with statistically significant reductions in all-cause mortality, neither IVUS (OR: 0.70 [95% CrI: 0.45 to 1.32]) nor OCT (OR: 0.81 [95% CrI: 0.47 to 1.59]) were associated with statistically significant reductions in target vessel failure, neither IVUS (OR: 0.88 [95% CrI: 0.43 to 2.44]) nor OCT (OR: 0.81 [95% CrI: 0.37 to 2.04]) were associated with statistically significant reductions in target lesion failure, and neither IVUS (OR: 0.82 [95% CrI: 0.60 to 1.06]) nor OCT (OR: 0.84 [95% CrI: 0.59 to 1.19]) were associated with statistically significant reductions in myocardial infarction.

**Conclusion:**

Intravascular imaging-guided, including IVUS and OCT, improved the postinterventional outcomes of PCI, notably suggesting their advantage over traditional angiography with no significant difference between IVUS and OCT.

**Supplementary Information:**

The online version contains supplementary material available at 10.1186/s12872-024-04105-5.

## Introduction

Percutaneous coronary intervention (PCI) has become one of the most commonly performed interventional life-saving procedures worldwide. It is now the dominant method for coronary revascularization, allowing pre-, per, and post-interventional assessment of coronary vascularization [1]. Yet, it has a few disadvantages in efficacy, such as the 2D aspect of the angiographic views and the inability to precisely measure the stenosis due to the X-ray source, the image intensifier, and the chemical properties of the cinefilm [2, 3]. Moreover, it is exposed to several safety risks related to its radiologically invasive nature and the chemotoxic or anaphylactoid effects of the iodinated contrast product [4].

Two primary modalities are currently being evaluated as adjunctive tools for PCI, including intravascular ultrasound (IVUS) and optical coherence tomography (OCT). IVUS has the advantage of providing detailed guidance on PCI at the pre-interventional time by characterizing the nature of the atherosclerotic plaque and the mechanism of stenosis along with thrombotic plaque morphology, lesion length, and reference vessel diameter. Moreover, it has a post-interventional advantage by assessing coronary stent implantation results, including minimal stent area and expansion [5]. These benefits had clinical implications as the use of IVUS guidance during PCI was correlated with a significant reduction in the risk of 3-year target lesion failure, medium-term mortality, and target vessel revascularization [6, 7]. Additionally, registry-based data revealed reduced flow-impairing coronary dissection rates among patients undergoing PCI with IVUS on an elective basis [8]. On the other hand, OCT produces a more sophisticated visualization of the coronary artery wall and microstructures via near-infrared light to produce high-definition, cross-sectional 3D volumetric images [9]. It has a shorter wavelength compared to IVUS (1.3 μm vs. ~ 40 μm at 40 MHz), which allows greater axial resolution (10–20 μm versus 50–150 μm) [9]. Indeed, real-world data showed that OCT optimized PCI outcomes, particularly during the complex left main and bifurcation lesions [10]. It further revealed reduced risks of major adverse cardiovascular events (MACE), myocardial infarction, or repeat revascularization when PCI is assisted by OCT [11].

However, the superiority of OCT-guided PCI or IVUS to angiography-guided PCI remains uncertain, especially with continuously updated evidence. In this systematic review and meta-analysis, we examined the available data from randomized controlled trials (RCTs), comparing the efficacy and safety of PCI directed by OCT, IVUS, or angiography.

## Methodology

### Protocol registration

We prospectively registered this network meta-analysis in the International Prospective Register of Systematic Reviews (PROSPERO) under ID: CRD42024507821. We conducted this network meta-analysis in accordance with the PRISMA and PRISMA NMA statement guidelines for systematic reviews and meta-analysis [12, 13] and the Cochrane Handbook for Systematic Reviews and Meta-Analysis guidelines [14].

### Data sources & search strategy

We systematically searched the following databases: Web of Science, SCOPUS, EMBASE, PubMed, and Cochrane Central Register of Controlled Trials (CENTRAL) up to September 2023. The detailed search strategy and results are shown in (Table S1).

### Eligibility criteria

We included RCTs with the following PICO criteria: population (P): Patients undergoing PCI; intervention (I): IVUS or OCT; control (C): coronary angiography; and outcomes (O): primary outcomes: major adverse cardiovascular events (MACE), while our secondary outcomes included: all-cause mortality, cardiac death, target vessel failure, target lesion failure, myocardial infarction, any revascularization, target vessel revascularization, stent thrombosis, CABG, and restenosis. Single-arm, observational studies, abstracts, and non-randomized trials were excluded.

### Study selection

After duplicates removal using Covidence software, six investigators (U.K., M.T., H.E., M.M.E., M.E., and A.K.E.) independently assessed the titles and abstracts of the retrieved records. Then, they screened the full texts in accordance with the previously mentioned eligibility criteria. Any disagreements were resolved via discussion.

### Data extraction

Using an Excel sheet, six reviewers (U.K., M.T., H.E., M.M.E., M.E., and A.K.E.) independently extracted summary characteristics of the included studies (study design, countries, total participants, intervention details (IVUS, OCT, and coronary angiography), MACE definition, follow-up period, and primary outcome), patients baseline characteristics (number of patients in each group, mean of age, male percentage, body mass index (BMI), left ventricular ejection fraction (LVEF), and comorbidities), and efficacy sheet (MACE, all-cause mortality, cardiac death, target vessel failure, target lesion failure, myocardial infarction, any revascularization, target vessel revascularization, stent thrombosis, CABG, and restenosis). Any disagreements were resolved through discussion.

### Risk of bias and certainty of evidence

Using the revised Cochrane collaboration’s tool for assessing the risk of bias in randomized trials (ROB 2) [15], six reviewers (U.K., M.T., H.E., M.M.E., M.E., and A.K.E.) independently assessed the included RCTs for risk of bias in domains that include the randomization process, deviations from intended interventions, missing outcome data, measurement of the outcome, selection of the reported result, and overall bias. Any disagreements were resolved via discussion.

### Statistical analysis

We conducted a Bayesian network meta-analysis using the “bnma” package on R, version 4.2.0. We will use a random-effects model to account for between-study variation in treatment effects for outcomes reported by a sufficient number of studies. We primarily described heterogeneity using tau, an absolute measure that represents the standard deviation of treatment effects across studies. Effect sizes were presented as odds ratios with accompanying 95% credible intervals. A league table was constructed to compare all treatments, and the Surface Under the Cumulative Ranking Area (SUCRA) provides a single-number summary. Additionally, we performed a frequentist sensitivity analysis to ensure that the robustness of our findings was not sensitive to the statistical framework adopted. This analysis was performed using both random-effects and fixed-effect models to ensure that our findings were robust to the approach to heterogeneity. Funnel plots were used to assess publication bias. When the number of studies permitted (minimum of 10), we formally assessed funnel plot asymmetry using Egger’s test (a linear regression test of asymmetry).

## Results

### Search results and study selection

**Fig. 1:**
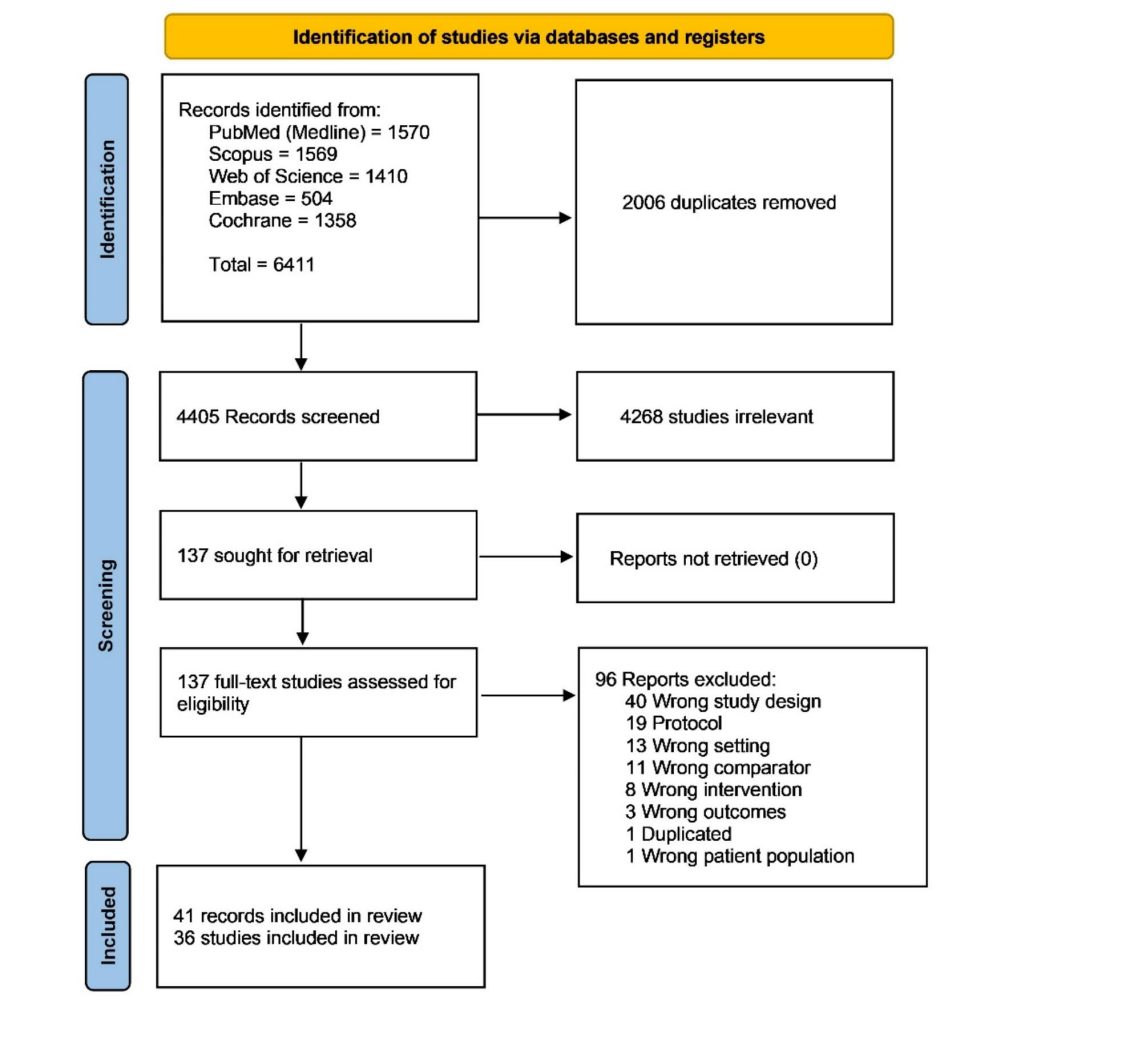
PRISMA flow chart of the screening process

Our search strategy resulted in 6,411 records from the previously mentioned databases. After removing duplicates, 4,405 records were included in the title and abstract screening, followed by 137 records in full-text screening. Finally, 41 publications (36 main records of the RCTs and five follow-up papers of some of the included RCTs) were included in our network meta-analysis (Fig. 1).

### Characteristics of included studies

Forty-one records (36 RCTs) were included [16–56], with 17,572 patients included, of whom 6,523 patients were in the IVUS group, 4,157 patients in the OCT group, and 6,892 patients in the coronary angiography group. A total of 21 RCTs compared IVUS with angiography [16, 18, 19, 22–24, 28–30, 33, 35–37, 40, 42–44, 46, 47, 49–52, 54–56], 12 RCTs compared OCT with angiography [16, 17, 21, 25, 26, 28, 31, 34, 38, 39, 48, 50, 53], and six RCTs compared IVUS with OCT [16, 20, 27, 28, 32, 41, 50]. Summary RCTs characteristics and baseline characteristics of the participants are shown in (Tables 1 and 2).

**Table 1:** Summary characteristics of the included RCTs

Study	Study design	Country	Total participants	Intervention	Control	MACE definition	Stent type	Primary outcome	Follow-up duration	
**Ali et al. 2016**,** Ali et al. 2021 (ILUMIEN III)**[16, 17]	Prospective, multi-center, single-blind, RCT	USA, Belgium, Germany, Italy, Japan, Netherlands, Spain, and UK	450	OCT-guided PCI and IVUS-guided PCI	Angiography-guided PCI	Composite of death, MI, stent thrombosis, or repeat revascularisation	DES	Final minimum stent area (MSA)	One year.	
**Ali et al. 2023 (ILUMIEN IV)**[18]	Prospective, multi-center, single-blind, RCT	Australia, Belgium, Canada, Denmark, France, Germany, Hong Kong, India, Italy, Japan, Netherlands, New Zealand, Portugal, Singapore, Spain, Sweden, Switzerland, Taiwan, UK, USA	2487	OCT-guided PCI	Angiography-guided PCI	NA	DES	Target-vessel failure	Two years	
**Antonsen et al. 2015 (OCTACS)**[19]	Prospective, single-center, RCT	Denmark	100	OCT-guided PCI	Angiography-guided PCI	NA	DES	Assess the percentage of uncovered struts and the presence of acute malapposition	Six months.	
**Chamié et al. 2021 (The iSIGHT)**[20]	Prospective, single-center, RCT	Brazil	150	OCT-guided PCI and IVUS-guided PCI	Angiography-guided PCI	Composite of cardiac death, nonfatal MI, and target lesion revascularization.	DES	The non-inferiority of postprocedure stent expansion, defined as minimum stent area (MSA) divided by the average lumen area of the distal and proximal references	Two years	
**Chieffo et al. 2013 (AVIO)**[21]	Prospective, multi-center, RCT	UK	284	IVUS-guided PCI	Angiography-guided PCI	Composite of any MI, cardiac death, and target vessel revascularization (TVR).	DES	Post-procedural in-lesion minimum lumen diameter (MLD) was evaluated using core laboratory quantitative coronary angiography (QCA).	Two years	
**Fallesen et al. 2022 (HONEST)**[22]	Prospective, single-center, RCT	Denmark	75	OCT-guided PCI	Angiography-guided PCI	NA	BVS	Rate of in-scaffold late lumen loss (LLL) at six months	Six months.	
**Frey et al. 2000 (SIPS)**[23]	Prospective, single-center, RCT	Germany	269	ICUS-Guided Group	Angio-Guided Group	Composite of all revascularization procedures (re-PTCA and CABG), myocardial infarction, and deaths	BMS	6-month angiographic minimal lumen diameter (MLD)	Two years	
**Zhang et al. 2018**,** Gao et al. 2021 (ULTIMATE)**[24, 56]	Prospective, multi-center, RCT	China	1448	IVUS-guided PCI	Angiography-guided PCI	NA	DES	Occurrence of TVF at three years after the index procedure, which included cardiac death, target vessel MI (TVMI), and clinically driven target vessel revascularization (TVR)	Three years	
**Gaster et al. 2003 and 2009**[25, 26]	Prospective, single-center, RCT	Denmark	108	IVUS-guided PCI	Angiography-guided PCI	Composite of death, Q wave AMI, or revascularisation procedures.	BMS	MACE rate	2.5 years (0.6–3.8 years, 25th and 75th centiles).	
**Gil et al. 2007 (DIPOL)**[27]	Prospective, multi-center, RCT	Poland	259	IVUS-guided PCI	Angiography-guided PCI	Composite of death, myocardial infarction, and repeat coronary revascularization [RCR]) that occurred at six months.	BMS	MACE rate	Six months.	
**Habara et al. 2012**[28]	Prospective, single-center, RCT	Japan	70	IVUS-guided PCI	OCT-guided PCI	NA	DES and BMS	Stent expansion was analyzed by IVUS.	NA	
**Holm et al. 2023 (OCTOBER)**[29]	Prospective, multi-center, RCT	Denmark	1201	OCT-guided PCI	Angiography-guided PCI	Composite of death from a cardiac cause, target-lesion myocardial infarction, or ischemia-driven target-lesion revascularization at a median follow-up of 2 years.	DES	MACE rate	Two years	
**Hong et al. 2015 and 2020 (The IVUS-XPL)**[30, 31]	Prospective, multi-center, RCT	Korea	1400	IVUS-guided PCI	Angiography-guided PCI	Composite of cardiac death, target lesion-related myocardial infarction, or ischemia-driven target lesion revascularization at one year	DES	MACE rate	Five years	
**Jakabcin et al. 2010 (HOME DES IVUS)**[32]	Prospective, single-center, RCT	Czech	210	IVUS-guided PCI	Angiography-guided PCI	Composite of death, myocardial infarction (MI), and target lesion revascularization (TLR)	DES	NA	18 months.	
**Jia et al. 2022 (EROSION III)**[33]	Prospective, multi-center, RCT	China	226	OCT-guided PCI	Angiography-guided PCI	Composite of cardiac death, Recurrent MI, TLR, malignant arrhythmia, unstable angina-induced rehospitalization, and stroke;	BVS	Patient-level rate of stent implantation, cardiac death, recurrent MI, TLR, and unstable angina-induced rehospitalization within one month.	One year	
**Kala et al. 2018 (ROBUST)**[34]	Prospective, multi-center, RCT	Czech	201	OCT-guided PCI	Angiography-guided PCI	Composite of death, myocardial infarction [MI], and target lesion revascularization [TLR]	DES	MACE rate	Nine months	
**Kang et al. 2023 (OCTIVUS)**[35]	Prospective, multi-center, RCT	Korea	2008	IVUS-guided PCI	OCT-guided PCI	NA	DES	Target-vessel failure (a composite of death from cardiac causes, target-vessel myocardial infarction, or ischemia-driven target-vessel revascularization)	One year	
**Kim et al. 2013 (RESET)**[36]	Prospective, multi-center, RCT	Korea	543	IVUS-guided PCI	Angiography-guided PCI	Composite of cardiovascular death, myocardial infarction, target vessel revascularization, or stent thrombosis at one year following intervention	DES	MACE rate	One year	
**Kim et al. 2015 (CTO-IVUS)**[37]	Prospective, multi-center, RCT	Korea	402	IVUS-guided PCI	Angiography-guided PCI	Composite of cardiac death, myocardial infarction, or target-vessel revascularization, respectively. After 12-month follow-up	DES	Cardiac death.	One year	
**Kim et al. 2015**[38]	Prospective, single-center, RCT	Korea	101	OCT-guided PCI	Angiography-guided PCI	Composite of cardiac death, nonfatal myocardial infarction, or patients requiring target lesion revascularization.	DES	the percentage of uncovered struts in the 6-month follow-up OCT assessments.	One year	
**Kubo et al. 2017 (OPINION)**[39]	Prospective, multi-center, RCT	Japan	817	IVUS-guided PCI	OCT-guided PCI	Composite of cardiac death, myocardial infarction, or ischemia-driven target lesion revascularization	DES	Target vessel failure	One year	
**Lee et al. 2020**[40]	Prospective, multi-center, RCT	Korea	176	OCT-guided PCI	Angiography-guided PCI	NA	DES	minimal scaffold area < 5 mm2, residual area stenosis > 20%, percent ISA struts > 5%, major edge dissection, or scaffold disruption.	NA	
**Lee et al. 2023 (RENOVATE-COMPLEX-PCI)**[41]	Prospective, multi-center, RCT	Korea	1639	IVUS-guided PCI	Angiography-guided PCI	NA	DES	Target vessel failure	Two years.	
**Liu et al. 2018**[42]	Prospective, single-center, RCT	China	336	IVUS-guided PCI	Angiography-guided PCI	Composite of cardiac death, myocardial infarction (MI), and target vessel revascularization (TVR).	DES	MACE rate	One year	
**Mariani et al. 2014**,** Mariani et al. 2015 (MOZART)**[43, 44]	Prospective, single-center, RCT	Brazil	83	IVUS-guided PCI	Angiography-guided PCI	NA	DES	the total volume contrast, Cardiovascular events agent used during PCI.	One year	
**Meneveau et al. 2016 (DOCTORS)**[45]	Prospective, multi-center, RCT	France	240	OCT-guided PCI	Angiography-guided PCI	NA	BMS/DES	Fractional flow reserve (FFR)	Six months	
**Mudra et al. 2001 (OPTICUS)**[46]	Prospective, multi-center, RCT	Germany, Spain, Sweden, Italy, Greece, France, Netherlands, United Kingdom, Belgium, and Israel	550	ICUS-guided PCI	Angiography-guided PCI	Composite of death, myocardial infarction, coronary bypass surgery, and repeat percutaneous intervention	BMS	The incidence of angiographic restenosis (0.50% lumen diameter reduction), minimal lumen diameter, and percent diameter stenosis after 6 months.	One year	
**Muramatsu et al. 2020 (MISTIC-1)**[47]	Prospective, multi-center, RCT	Japan	109	IVUS-guided PCI	OCT-guided PCI	Composite of cardiovascular mortality, target-vessel myocardial infarction, or clinically driven target-lesion revascularization.	DES	in-segment minimum lumen area assessed using OFDI at eight months and MACE rate	Three years	
**Oemrawsingh et al. 2003 (TULIP)**[48]	Prospective, single-center, RCT	Netherlands	150	IVUS-guided PCI	Angiography-guided PCI	NA	BMS	6-month minimal lumen diameter (MLD) and the combined end point of death, myocardial infarction, and target-lesion revascularization (TLR).	Six months	
**Russo et al. 2009 (AVID)**[49]	Prospective, multi-center, RCT	USA	800	IVUS-guided PCI	Angiography-guided PCI	NA	BMS	target lesion revascularization at 12 months	One year	
**Schiele et al. 1998 (The RESIST)**[50]	Prospective, multi-center, single-blind, RCT	France	155	IVUS-guided PCI	Angiography-guided PCI	NA	BMS	the 6-month restenosis rate, defined as 0.50% narrowing at the stent site or 5 mm proximal or distal to the stent, as assessed by QCA	Six months.	
**Schneider et al. 2021 (OPTICO‑integration II)**[51]	Prospective, single-center, RCT	Germany	56	OCT-guided PCI	Angiography-guided PCI	NA	NA	composite imaging endpoint, including major edge dissections and/or LGM as assessed by post-procedural OCT	NA	
**Tan et al. 2015**[52]	Prospective, single-center, RCT	China	123	IVUS-guided PCI	Angiography-guided PCI	Composite of death, non-fatal myocardial infarction, and target lesion revascularization (TLR).	DES	MACE rate	Two years	
**Tian et al. 2015 (AIR-CTO)**[53]	Prospective, multi-center, RCT	China	230	IVUS-guided PCI	Angiography-guided PCI	NA	DES	late lumen loss (LLL) at 12 months	Two years	
**Ueki et al. 2020 (OPTICO BVS)**[54]	Prospective, multi-center, RCT	Switzerland	38	OCT-guided PCI	Angiography-guided PCI	NA	BVS	in-scaffold minimal lumen area (MLA) at 6-month	One year	
**Wang et al. 2015**[55]	Prospective, single-center, RCT	China	80	IVUS-guided PCI	Angiography-guided PCI	Composite of refractory myocardial ischemia, second target vessel reconstruction, new AMI, and cardiac death.	NA	MACE rate	One year	

MACE: major adverse cardiovascular events, DES: drug-eluting stent, BVS: Bioresorbable vascular scaffold, BMS: Bare-metal stent, PCI: Percutaneous coronary intervention, IVUS: Intravascular ultrasound, OCT: Optical coherence tomography, NA: Not available

**Table 2:** Baseline characteristics of the participants

Study ID	Study arm	Number of patients in each group	Age (Years) Mean (SD)	Gender (Male) N. (%)	BMI, Mean (SD)	LVEF, Mean (SD)	Comorbidities N. (%)				
							Dyslipidemia	Hypertension	Smoking	Diabetes	MI
**Ali et al. 2016**,** Ali et al. 2021 (ILUMIEN III)**[16, 17]	**IVUS**	146	66.33 (8.23)	107 (73)	27.8 (4.2)	NA	107 (73)	113 (77)	19 (13)	55 (38)	29 (20)
	**OCT**	158	65.6 (9.72)	109 (69)	28.2 (4.86)	NA	115 (73)	124 (78)	28 (18)	52 (33)	35 (22)
	**Angiography**	146	65.6 (13.47)	107 (73)	27.8 (4.64)	NA	112 (77)	109 (75)	35 (24)	42 (29)	32 (22)
**Ali et al. 2023 (ILUMIEN IV)**[18]	**OCT**	1233	65.5 (10.5)	968 (78.5)	28.7 (5.3)	55.2 (8.6)	808 (65.5)	880 (71.4)	242 (19.6)	523 (42.4)	252 (20.4)
	**Angiography**	1254	65.7 (10.3)	956 (76.2)	28.8 (5.5)	55.2 (8.7)	860 (68.6)	928 (74.0)	247 (19.7)	521 (41.5)	303 (24.2)
**Antonsen et al. 2015 (OCTACS)**[19]	**OCT**	50	61.8 (9.4)	36 (72.0)	NA	NA	22 (44.0)	28 (56.0)	23 (46.0)	8 (16.0)	2 (4.0)
	**Angiography**	50	62.6 (11.0)	34 (68.0)	NA	NA	19 (38.0)	28 (56.0)	18 (36.0)	5 (10.0)	0 (0.0)
**Chamié et al. 2021 (The iSIGHT)**[20]	**IVUS**	50	59.32 (10.37)	36 (72.0)	26.96 (4.62)	NA	30 (60.0)	42 (84.0)	14 (28.0)	20 (40.0)	17 (34.0)
	**OCT**	51	59.92 (8.92)	31 (60.8)	28.58 (4.16)	NA	36 (70.6)	46 (90.2)	17 (33.3)	17 (33.3)	15 (29.4)
	**Angiography**	49	58.59 (10.20)	38 (77.5)	28.81 (5.06)	NA	28 (57.2)	39 (79.6)	14 (28.6)	22 (44.9)	17 (34.7)
**Chieffo et al. 2013 (AVIO)**[21]	**IVUS**	142	63.9 (10.1)	117 (82.3)	NA	55.3 (8.5)	100 (70.4)	101 (70.4)	49 (34.5)	34 (23.90	NA
	**Angiography**	142	63.6 (11.0)	109 (767.)	NA	55.9 (8.6)	109 (76.8)	95 (66.9)	44 (31)	38 (26.8)	NA
**Fallesen et al. 2022 (HONEST)**[22]	**OCT**	37	61.1 (10.9)	28 (78.4)	28.8 (4.5)	NA	15 (40.5)	15 (40.5)	10 (27.0)	2 (5.4)	4 (10.8)
	**Angiography**	38	61.7 (10.1)	31 (81.6)	28.3 (4.3)	NA	13 (34.2)	16 (42.1)	12 (31.6)	4 (10.5)	4 (10.5)
**Frey et al. 2000 (SIPS)**[23]	**ICUS**	121	61.2 (8.1)	99 (82)	NA	0.83 (0.72)	105 (88)	77 (64)	56 (47)	19 (16)	69 (58)
	**Angiography**	148	60.7 (9.6)	113 (76)	NA	0.70 (0.69)	129 (87)	82 (56)	66 (45)	24 (16)	77 (52)
**Zhang et al. 2018**,** Gao et al. 2021 (ULTIMATE)**[24, 56]	**IVUS**	724	65.2 (10.9)	535 (73.9)	25.3 (18.0)	60.9 (7.9)	389 (53.7)	512 (70.7)	253 (34.9)	217 (30.0)	67 (9.3)
	**Angiography**	724	65.9 (9.8)	530 (73.2)	25.4 (19.3)	60.3 (9.3)	400 (55.2)	521 (72.0)	228 (31.5)	226 (31.2)	86 (11.9)
**Gaster et al. 2003 and 2009**[25, 26]	**IVUS**	54	56.6 (25.13)	54 (100)	NA	65 (12)	52 (96)	11 (20)	16 (30)	2 (4)	29 (54)
	**Angiography**	54	56 (34.27)	54 (100)	NA	69 (12)	50 (93)	12 (24)	8 (15)	9 (11)	24 (44)
**Gil et al. 2007 (DIPOL)**[27]	**IVUS**	179	53.8 (8.7)	131 (73.1)	NA	53 (8.5)	87 (48.6)	NA	87 (48.6)	21 (11)	74 (41.5)
	**Angiography**	80	54 (8)	58(73)	NA	48 (10)	32 (40)	NA	42 (52)	9 (11)	32 (40)
**Habara et al. 2012**[28]	**IVUS**	35	67.4 (8.0)	26 (74.3)	NA	NA	21 (60)	9 (25.7)	3 (8.6)	9 (25.7)	3 (8.6)
	**OCT**	35	67.6 (9.7)	29 (82.9)	NA	NA	15 (42.9)	11 (31.4)	7 (20.0)	11 (31.4)	3 (8.6)
**Holm et al. 2023 (OCTOBER)**[29]	**OCT**	600	66.4 (10.5)	473 (78.8)	28.0 (4.6)	56.5 (7.43)	456 (76.0)	422 (70.3)	77 (12.8)	103 (17.2)	170 (28.3)
	**Angiography**	601	66.2 (9.9)	475 (79)	28.2 (4.9)	56 (7.43)	471 (78.4)	448 (74.5)	85 (14.1)	97 (16.1)	180 (30.0)
**Hong et al. 2015 and 2020 (The IVUS-XPL)**[30, 31]	**IVUS**	700	64 (9)	483 (69)	24.6 (3.0)	62.9 (9.8)	471 (67)	454 (65)	155 (22)	250 (36)	34 (5)
	**Angiography**	700	64 (9)	481 (69)	24.8 (3.1)	62.4 (10.2)	458 (65)	444 (63)	181 (26)	256 (37)	29 (4)
**Jakabcin et al. 2010 (HOME DES IVUS)**[32]	**IVUS**	105	60.2 (11)	75 (71)	NA	NA	69 (66)	75 (71)	37 (35)	47 (45)	34 (32)
	**Angiography**	105	59.4 (13)	77 (73)	NA	NA	66 (63)	70 (67)	42 (40)	44 (42)	39 (37)
**Jia et al. 2022 (EROSION III)**[33]	**OCT**	112	54.5 (11.2)	89 (79.5)	NA	NA	NA	47 (42.0)	64 (57.1)	29 (25.9)	NA
	**Angiography**	114	56.4 (10.4)	91 (79.8)	NA	NA	NA	45 (39.5)	73 (64.0)	19 (16.7)	NA
**Kala et al. 2018 (ROBUST)**[34]	**OCT**	105	57 (6.9)	92 (83)	NA	NA	NA	53 (50)	67 (64)	18 (17)	11 (1)
	**Angiography**	96	59 (6.2)	84 (87)	NA	NA	NA	50 (52)	54 (59)	25 (26)	58 (6)
**Kang et al. 2023 (OCTIVUS)**[35]	**IVUS**	1003	65.1 (10.5)	787 (78.3)	25 (3.1)	60.1 (7.5)	841 (83.9)	639 (63.7)	189 (18.8)	345 (34.4)	63 (6.3)
	**OCT**	1005	64.3 (10.3)	788 (78.6)	24.9 (3.2)	60.5(7.2)	840 (83.6)	647 (64.4)	217 (21.6)	325 (32.3)	78 (7.8)
**Kim et al. 2013 (RESET)**[36]	**IVUS**	269	62.8 (9.30)	177 (65.8)	NA	55.3 (23.9)	165 (61.3)	165 (61.3)	67 (22.6)	190 (64)	3 (1.0)
	**Angiography**	274	64.3 (8.7)	150 (54.7)	NA	54 (25)	165 (61.7)	178 (65.8)	38 (15.4)	144 (58.5)	8 (3.3)
**Kim et al. 2015 (CTO-IVUS)**[37]	**IVUS**	201	61 (11.1)	162 (80.6)	NA	56.9 (13.1)	NA	126 (62.7)	71 (35.5)	70 (34.8)	16 (8)
	**Angiography**	201	61.4 (10.1)	162 (80.6)	NA	56.7 (11.4)	NA	128 (63.7)	69 (34.3)	68 (33.8)	16 (8)
**Kim et al. 2015**[38]	**OCT**	50	58.8 (10.8)	39 (78)	NA	64.2 (7.4)	33 (66)	27 (54)	16 (32)	16 (32)	3 (6)
	**Angiography**	51	61.6 (9.7)	37 (72.5)	NA	63.8 (8.6)	37 (72.5)	25 (49)	15 (29.4)	16 (31.4)	8 (2)
**Kubo et al. 2017 (OPINION)**[39]	**IVUS**	405	68 (9)	322 (79.5)	NA	NA	321 (79.3)	299 (73.8)	73 (18)	165 (40.7)	61 (15.1)
	**OCT**	412	69 (9)	315 (76.5)	NA	NA	316 (76.7)	315 (76.5)	67 (16.3)	169 (41)	70 (17)
**Lee et al. 2020**[40]	**OCT**	88	57.8 (10.4)	64 (72.7)	24.8 (3.2)	65 (5.5)	73 (83.0)	51 (58.0)	18 (20.5)	18 (20.5)	6 (6.8)
	**Angiography**	88	59.5 (8.9)	67 (76.1)	25.1 (2.7)	67 (6)	77 (87.5	56 (63.6)	22 (25.0)	25 (28.4)	6 (6.8)
**Lee et al. 2023 (RENOVATE-COMPLEX-PCI)**[41]	**IVUS**	1092	65.3 (10.3)	869 (79.6)	NA	58.4 (11.9)	560 (51.3)	682 (62.5)	212 (19.4)	394 (36.1)	75 (6.9)
	**Angiography**	547	66 (10)	431 (78.8)	NA	59.3 (11)	280 (51.2)	323 (59)	95 (17.4)	223 (40.8)	42 (7.7)
**Liu et al. 2018**[42]	**IVUS**	167	65.3 (10.6)	106 (63.5)	23.8 (3.8)	55.6 (11.7)	63 (37.7)	116 (69.5)	62 (37.1)	56 (33.5)	29 (17.4)
	**Angiography**	169	64.9 (11.2)	108 (63.9)	24.1 (2.9)	58.4 (10.5)	64 (37.9)	122 (72.2)	60 (35.5)	52 (30.8)	24 (14.2)
**Mariani et al. 2014**,** Mariani et al. 2015 (MOZART)**[43, 44]	**IVUS**	41	67.1 (4.4)	25 (61)	NA	NA	NA	40 (97.6)	17 (41.5)	30 (73.2)	NA
	**Angiography**	42	62.1 (4.8)	24 (57.1)	NA	NA	NA	42 (100)	17 (40.4)	34 (81)	NA
**Meneveau et al. 2016 (DOCTORS)**[45]	**OCT**	120	60.8 (11.5)	95 (79.2)	NA	NA	59 (49.2)	67 (55.8)	47 (39.2)	26 (21.7)	NA
	**Angiography**	120	60.2(11.3)	91 (75.8)	NA	NA	56 (46.7)	50 (41.7)	51 (42.5)	19 (15.8)	NA
**Mudra et al. 2001 (OPTICUS)**[46]	**IVUS**	273	60.1 (10)	77 (28.2)	NA	56.5 (14)	NA	131 (48)	188 (69)	46 (17)	87 (32)
	**Angiography**	275	61.5 (9.5)	78 (28.3)	NA	57.7 (14.3)	NA	143 (52)	181 (66)	46 (17)	87 (32)
**Muramatsu et al. 2020 (MISTIC-1)**[47]	**IVUS**	55	71 (8)	44 (80)	NA	57 (12)	36 (65.5)	39 (70.9)	12 (21.8)	24 (43.6)	16 (29.1)
	**OCT**	54	72 (9.5)	41 (75.9)	NA	58 (11)	43 (79.6)	34 (63)	22 (40.7)	27 (50)	19 (35.2)
**Oemrawsingh et al. 2003 (TULIP)**[48]	**IVUS**	74	61 (10)	71 (95.9)	NA	0	NA	27 (36.4)	40 (54)	16 (21.6)	NA
	**Angiography**	76	63 (10)	72 (94.7)	NA	0	NA	30 (39.4)	53 (69.7)	21 (27.6)	NA
**Russo et al. 2009 (AVID)**[49]	**IVUS**	394	62 (12)	288 (73)	NA	53 (13)	158 (40)	181 (46)	NA	59 (15)	138 (35)
	**Angiography**	406	63 (11)	276 (68)	NA	55 (13)	179 (44)	183 (45)	NA	69 (17)	118 (29)
**Schiele et al. 1998 (The RESIST)**[50]	**IVUS**	79	57 (10)	68 (86)	NA	53 (13)	NA	24 (30)	55 (70)	9 (11)	54 (68)
	**Angiography**	76	56 (12)	71 (93)	NA	51 (9)	NA	26 (34)	51 (67)	8 (11)	48 (63)
**Schneider et al. 2021 (OPTICO‑integration II)**[51]	**OCT**	28	NA	NA	NA	NA	NA	NA	NA	NA	NA
	**Angiography**	28	NA	NA	NA	NA	NA	NA	NA	NA	NA
**Tan et al. 2015**[52]	**IVUS**	61	76.54 (4.95)	38 (62.2)	NA	55.32 (5.02)	NA	25 (41)	27 (44.3)	21 (34.4)	10 (16.4)
	**Angiography**	62	75.85 (3.49)	43 (69.3)	NA	53.33 (7.14)	NA	29 (46.8)	29 (46.8)	18 (29.5)	13 (21)
**Tian et al. 2015 (AIR-CTO)**[53]	**IVUS**	115	NA	NA	NA	NA	NA	NA	NA	NA	NA
	**Angiography**	115	NA	NA	NA	NA	NA	NA	NA	NA	NA
**Ueki et al. 2020 (OPTICO BVS)**[54]	**OCT**	19	63.3 (12.7)	15 (79)	27.7 (4.1)	61 (7.6)	13 (68)	7 (37)	7 (37)	4 (21)	4 (21)
	**Angiography**	19	62.9 (9.1)	15 (79)	28.2 (3.7)	64.4 (10.5)	12 (63)	11 (58)	6 (32)	4 (21)	2 (11)
**Wang et al. 2015**[55]	**IVUS**	38	56.4 (9.4)	23 (60.5)	NA	48.3 (5.7)	NA	15 (39.5)	19 (50)	8 (21.1)	NA
	**Angiography**	42	53.7 (11.8)	28 (66.7)	NA	49.7 (5.9)	NA	10 (23.8)	25 (59.5)	5 (11.9)	NA

SD, standard deviation; BMI, body mass index; LVEF: left ventricular ejection fraction; MI: Myocardial infarction; IVUS: Intravascular ultrasound; OCT: Optical coherence tomography; NA: not available

### Risk of bias and certainty of evidence

**Fig. 2:**
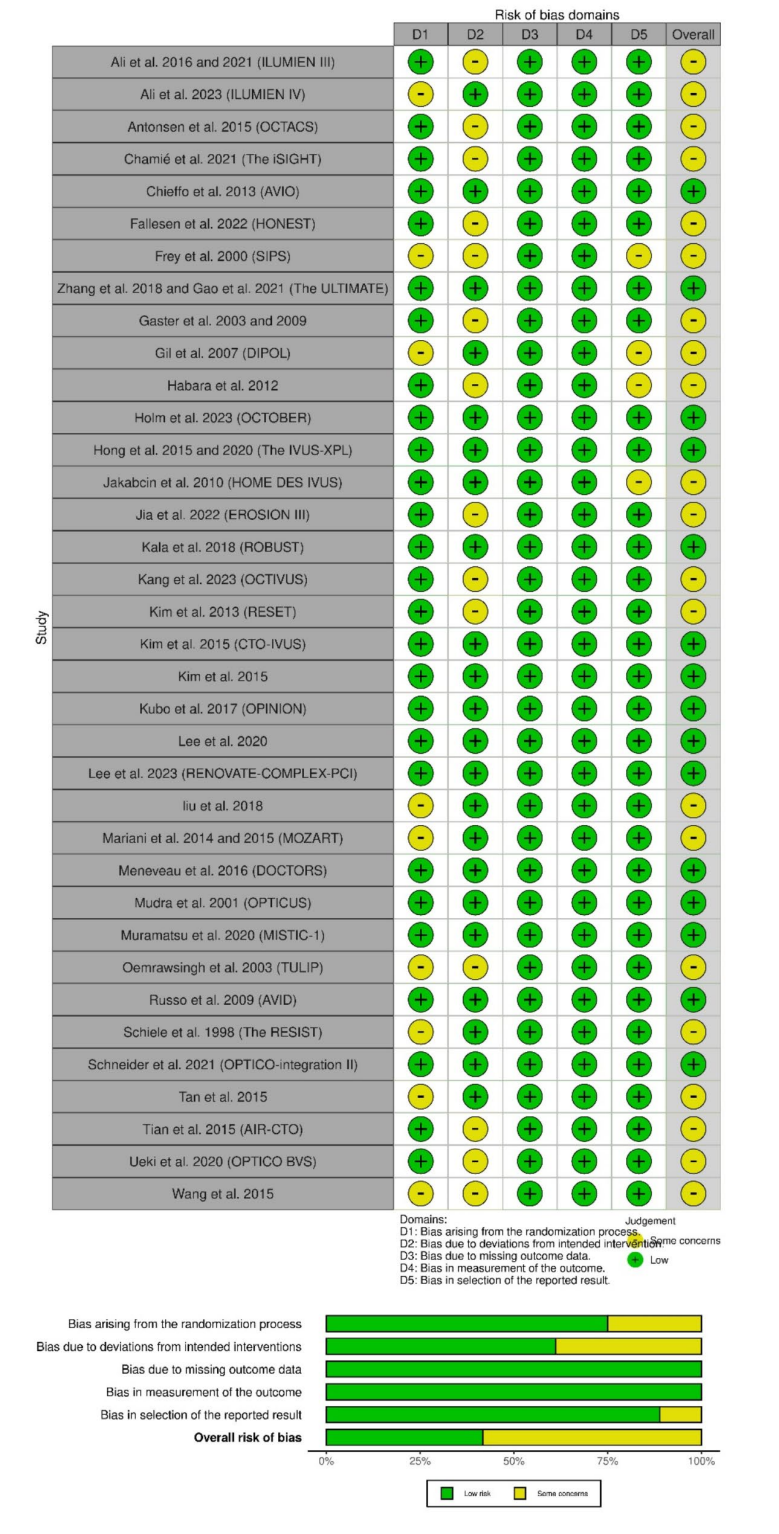
Quality assessment of risk of bias in the included trials. The upper panel presents a schematic representation of risks (low = green, unclear = yellow, and high = red) for specific types of biases of each study in the review. The lower panel presents risks (low = green, unclear = yellow, and high = red) for the subtypes of biases of the combination of studies included in this review

ROB 2.0 assessment showed that 15 RCTs had an overall low risk of bias; however, 21 RCTs had some concerns due to concerns about the randomization process, deviations from the interventions, and selection of the reported results (Fig. 2).

### Primary outcome: MACE

**Fig. 3:**
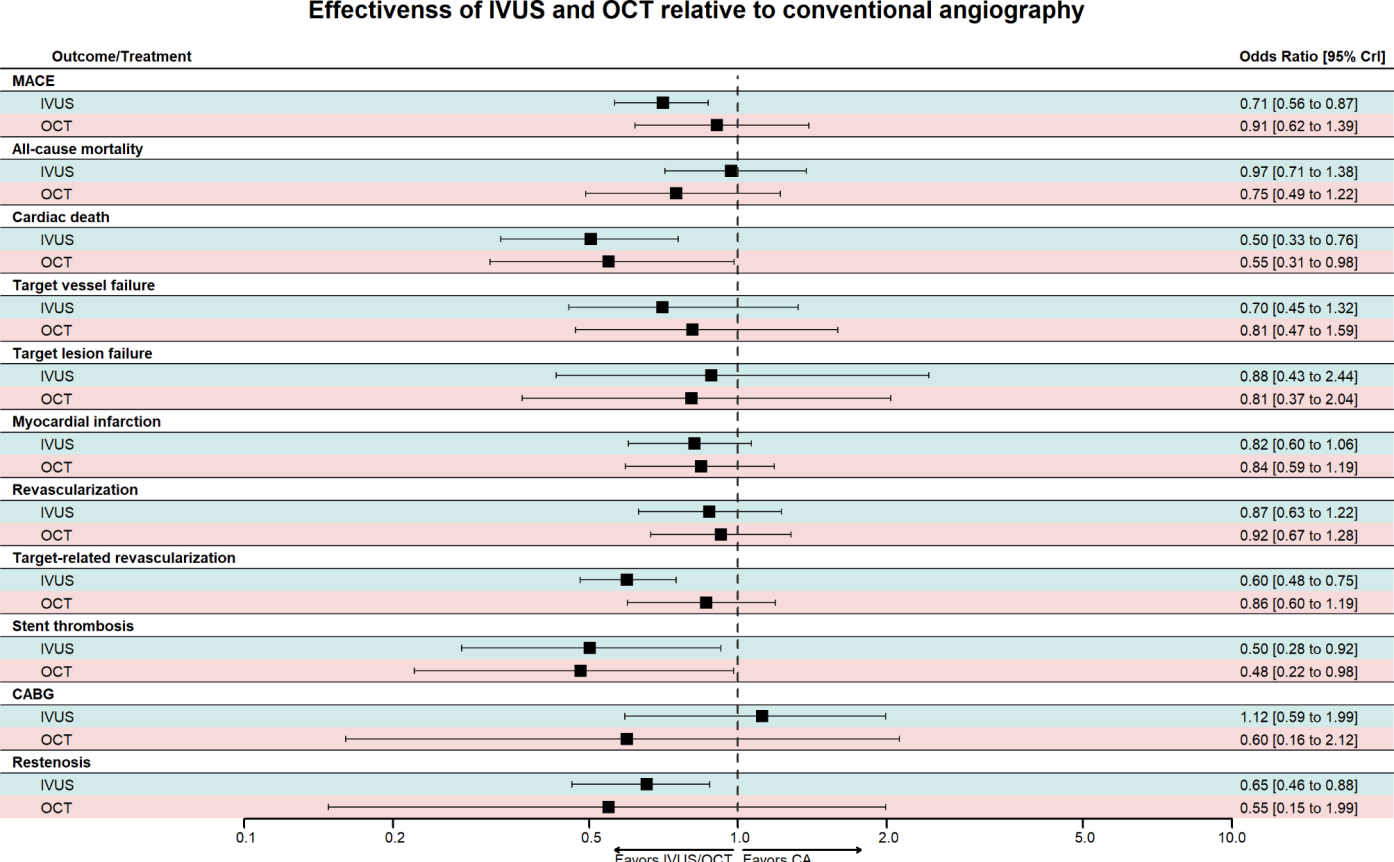
Forest plot of the clinical outcomes, OR: odds ratio, CI: confidence interval

**Fig. 4:**
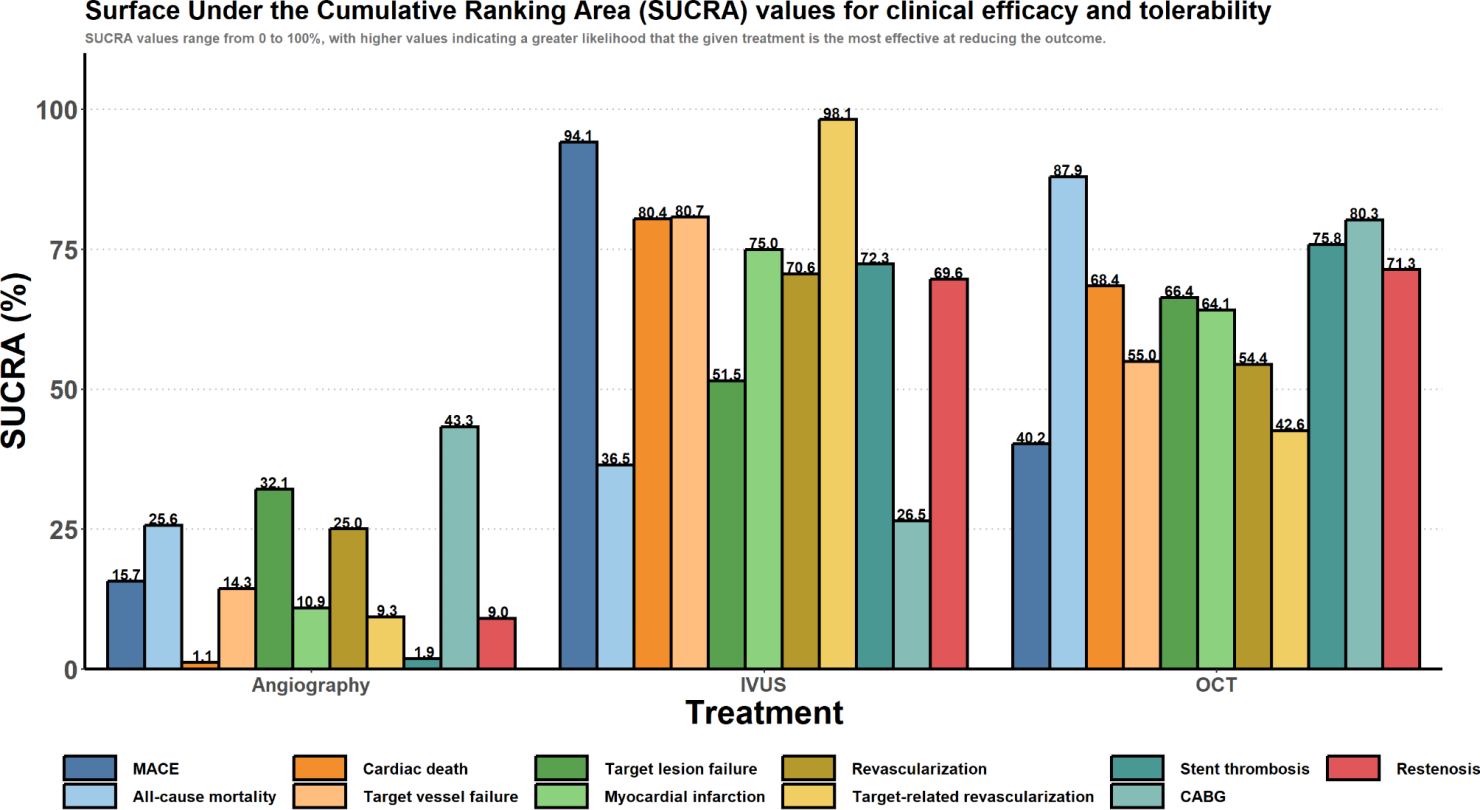
SUCRA analysis

Pooling 22 RCTs [16, 18, 19, 21–26, 28–32, 35, 39, 40, 43, 46, 47, 49, 50, 52, 54, 56], compared to conventional angiography, IVUS significantly reduced the risk of MACE (OR: 0.71 [95% CrI: 0.56 to 0.87]). Although rates of MACE were numerically lower with OCT compared to conventional angiography, this did not reach statistical significance (OR: 0.91 [95% CrI: 0.62 to 1.39]) **(**Fig. 3; Table 3). Based on the SUCRA analysis, IVUS had the highest probability of reducing revascularization (94.1%), followed by OCT (40.2%) and angiography (15.7%) (Fig. 4).

### Secondary outcomes

#### All-cause mortality

Pooling 26 RCTs [16–21, 24, 26–30, 32, 33, 36–43, 47, 48, 50, 51, 53–56], neither IVUS (OR: 0.97 [95% CrI: 0.71 to 1.38]) nor OCT (OR: 0.75 [95% CrI: 0.49 to 1.22]) were associated with statistically significant reductions in all-cause mortality compared to conventional angiography (Fig. 3; Table 3). Based on the SUCRA analysis, OCT had the highest probability of reducing all-cause mortality (87.9%), followed by IVUS (36.5%) and angiography (25.6%) (Fig. 4).

#### Cardiac death

Pooling 22 RCTs [16–19, 21–23, 25, 27–30, 32, 33, 35, 39, 41, 46, 47, 49–53, 55, 56], compared to conventional angiography, IVUS significantly reduced the risk of cardiac death (OR: 0.50 [95% CrI: 0.33 to 0.76]), as did OCT (OR: 0.55 [95% CrI: 0.31 to 0.98]) (Fig. 3; Table 3). Based on the SUCRA analysis, IVUS had the highest probability of reducing cardiac death (80.4%), followed by OCT (68.4%) and angiography (1.1%) (Fig. 4).

#### Target vessel failure

Target-vessel failure was defined as death from cardiac causes, target-vessel myocardial infarction, or ischemia-driven target-vessel revascularization. Upon pooling six RCTs [16, 17, 27, 28, 32, 33, 51, 55], neither IVUS (OR: 0.70 [95% CrI: 0.45 to 1.32]) nor OCT (OR: 0.81 [95% CrI: 0.47 to 1.59]) was associated with statistically significant reductions in target vessel failure compared to conventional angiography (Fig. 3; Table 3). Based on the SUCRA analysis, IVUS had the highest probability of reducing target vessel failure (80.7%), followed by OCT (55.0%) and angiography (14.3%) (Fig. 4).

#### Target lesion failure

Target-lesion failure was defined as death from cardiac causes, target-vessel myocardial infarction, or ischemia-driven target-lesion revascularization. Upon pooling four RCTs [16, 17, 27, 28, 51, 55], neither IVUS (OR: 0.88 [95% CrI: 0.43 to 2.44]) nor OCT (OR: 0.81 [95% CrI: 0.37 to 2.04]) was associated with statistically significant reductions in target lesion failure compared to conventional angiography (Fig. 3; Table 3). Based on the SUCRA analysis, OCT had the highest probability of reducing target lesion failure (66.4%), followed by IVUS (51.5%) and angiography (32.1%) (Fig. 4).

#### Myocardial infarction

Pooling 27 RCTs [16–21, 24–30, 32, 33, 35–38, 40–43, 46–50, 52, 56], compared to conventional angiography, neither IVUS (OR: 0.82 [95% CrI: 0.60 to 1.06]) nor OCT (OR: 0.84 [95% CrI: 0.59 to 1.19]) was associated with statistically significant reductions in myocardial infarction (Fig. 3; Table 3). Based on the SUCRA analysis, IVUS had the highest probability of reducing myocardial infarction (75.0%), followed by OCT (64.1%) and angiography (10.9%) (Fig. 4).

#### Any revascularization

Any revascularization is defined as any repeat revascularization (PCI or coronary artery bypass grafting). Upon Pooling 12 RCTs [16, 17, 19–21, 27, 28, 33, 35–37, 41, 48, 54], neither IVUS (OR: 0.87 [95% CrI: 0.63 to 1.22]) nor OCT (OR: 0.92 [95% CrI: 0.67 to 1.28]) were associated with statistically significant reductions in any revascularization compared to conventional angiography (Fig. 3; Table 3). Based on the SUCRA analysis, IVUS had the highest probability of reducing any revascularization (70.6%), followed by OCT (54.4%) and angiography (25.0%) (Fig. 4).

#### Target-vessel-related revascularization

Target-vessel-revascularization was defined as a target vessel requiring any repeat revascularization (PCI or coronary artery bypass grafting). Upon pooling 18 RCTs [16–19, 21, 27–30, 32, 33, 35–38, 41, 47, 48, 51, 52, 55, 56], compared to conventional angiography, IVUS significantly reduced the risk of target-vessel-related revascularization (OR: 0.60 [95% CrI: 0.48 to 0.75]). However, this was not seen with OCT (OR: 0.86 [95% CrI: 0.60 to 1.19]) (Fig. 3; Table 3). Based on the SUCRA analysis, IVUS had the highest probability of reducing target-vessel-related revascularization (98.1%), followed by OCT (42.6%) and angiography (9.3%) (Fig. 4).

#### CABG

CABG was defined as any repeat revascularization by coronary artery bypass grafting. Upon pooling nine RCTs [18, 19, 21, 27, 35, 40, 43, 47, 51, 55, 56], neither IVUS (OR: 1.12 [95% CrI: 0.59 to 1.99]) nor OCT (OR: 0.60 [95% CrI: 0.16 to 2.12]) were associated with statistically significant reductions in CABG operations compared to conventional angiography (Fig. 3; Table 3). Based on the SUCRA analysis, OCT had the highest probability of reducing CABG operations (80.3%), followed by angiography (43.3%) and IVUS (26.5%) (Fig. 4).

#### Stent thrombosis

Pooling 24 RCTs [16, 17, 19, 21–24, 26–33, 35–39, 41, 43, 46, 47, 50, 51, 53, 55], compared to conventional angiography, IVUS significantly reduced the risk of stent thrombosis (OR: 0.50 [95% CrI: 0.28 to 0.92]), as did OCT (OR: 0.48 [95% CrI: 0.22 to 0.98]) **(**Fig. 3**and** Table 3**)**. Based on the SUCRA analysis, OCT had the highest probability of reducing stent thrombosis (75.8%), followed by IVUS (72.3%) and Angiography (1.9%) **(**Fig. 4**)**.

**Table 3:** League table showing all possible comparisons in the network meta-analysis

Treatment	Angiography	IVUS	OCT
**MACE**			
**Angiography**	---	**0.71 (0.56 to 0.87)**	0.91 (0.62 to 1.39)
**IVUS**	**1.42 (1.15 to 1.78)**	---	1.28 (0.85 to 2.07)
**OCT**	1.10 (0.72 to 1.62)	0.78 (0.48 to 1.18)	---
**All-cause mortality**			
**Angiography**	---	0.97 (0.71 to 1.38)	0.75 (0.49 to 1.22)
**IVUS**	1.03 (0.73 to 1.40)	---	0.77 (0.48 to 1.32)
**OCT**	1.34 (0.82 to 2.03)	1.30 (0.76 to 2.10)	---
**Cardiac death**			
**Angiography**	---	**0.50 (0.33 to 0.76)**	**0.55 (0.31 to 0.98)**
**IVUS**	**1.99 (1.32 to 3.03)**	---	1.10 (0.57 to 2.15)
**OCT**	**1.83 (1.02 to 3.18)**	0.91 (0.46 to 1.74)	---
**Target vessel failure**			
**Angiography**	---	0.70 (0.45 to 1.32)	0.81 (0.47 to 1.59)
**IVUS**	1.42 (0.75 to 2.20)	---	1.15 (0.58 to 2.15)
**OCT**	1.24 (0.63 to 2.14)	0.87 (0.47 to 1.72)	---
**Target lesion failure**			
**Angiography**	---	0.88 (0.43 to 2.44)	0.81 (0.37 to 2.04)
**IVUS**	1.13 (0.41 to 2.33)	---	0.91 (0.32 to 2.19)
**OCT**	1.24 (0.49 to 2.74)	1.10 (0.46 to 3.11)	---
**Myocardial infarction**			
**Angiography**	---	0.82 (0.60 to 1.06)	0.84 (0.59 to 1.19)
**IVUS**	1.23 (0.94 to 1.67)	---	1.03 (0.70 to 1.58)
**OCT**	1.19 (0.84 to 1.69)	0.97 (0.63 to 1.43)	---
**Revascularization**			
**Angiography**	---	0.87 (0.63 to 1.22)	0.92 (0.67 to 1.28)
**IVUS**	1.14 (0.82 to 1.59)	---	1.06 (0.70 to 1.59)
**OCT**	1.08 (0.78 to 1.50)	0.95 (0.63 to 1.43)	---
**Target-related revascularization**			
**Angiography**	---	**0.60 (0.48 to 0.75)**	0.86 (0.60 to 1.19)
**IVUS**	**1.68 (1.33 to 2.09)**	---	1.45 (0.96 to 2.09)
**OCT**	1.16 (0.84 to 1.68)	0.69 (0.48 to 1.04)	---
**Stent thrombosis**			
**Angiography**	---	**0.50 (0.28 to 0.92)**	**0.48 (0.22 to 0.98)**
**IVUS**	**2.00 (1.08 to 3.63)**	---	0.96 (0.37 to 2.33)
**OCT**	**2.08 (1.02 to 4.52)**	1.04 (0.43 to 2.71)	---
**CABG**			
**Angiography**	---	1.12 (0.59 to 1.99)	0.60 (0.16 to 2.12)
**IVUS**	0.89 (0.50 to 1.69)	---	0.53 (0.13 to 2.09)
**OCT**	1.68 (0.47 to 6.24)	1.88 (0.48 to 7.58)	---
**Restenosis**			
**Angiography**	---	**0.65 (0.46 to 0.88)**	0.55 (0.15 to 1.99)
**IVUS**	**1.53 (1.14 to 2.17)**	---	0.84 (0.22 to 3.17)
**OCT**	1.83 (0.50 to 6.76)	1.19 (0.32 to 4.52)	---

IVUS: Intravascular ultrasound, OCT: Optical coherence tomography

Each cell shows the odds ratio and 95% credible interval comparing the intervention in the column heading versus the intervention in each row

#### Restenosis

Restenosis was defined as the percent diameter of stenosis at follow-up at ≥ 50%, confirmed by angiography. Upon pooling 12 RCTs [18, 19, 26, 32, 41–44, 47, 52–54, 56], compared to conventional angiography, IVUS significantly reduced the risk of restenosis (OR: 0.65 [95% CrI: 0.46 to 0.88]). Although rates of restenosis were numerically lower with OCT compared to conventional angiography, this did not reach statistical significance (OR: 0.55 [95% CrI: 0.15 to 1.99]) **(**Fig. 3**and** Table 3**)**. Based on the SUCRA analysis, OCT had the highest probability of reducing restenosis (71.3%), followed by IVUS (69.6%) and Angiography (9.0%) **(**Fig. 4**)**.

### Assessment of inconsistency and heterogeneity

Assessments of pairwise heterogeneity and inconsistency (assessed by comparing the direct and indirect estimates via a node-splitting approach) are shown in **(Table S3)**. There was no inconsistency or heterogeneity across any of the assessed outcomes.

### Sensitivity analysis and assessment of publication bias

Figures S1-S22 show the sensitivity frequentist analysis (under both random effects and a fixed effect). Figures S23-S33 show funnel plots used to assess publication bias.

## Discussion

The available body of evidence supports the superiority of IVUS and, to a lesser degree, OCT over angiography as imaging modalities to assist percutaneous recanalization among patients with coronary artery disease. A decrease in MACE, target-vessel-related revascularization, stent thrombosis, and restenosis risks were noted with IVUS but not OCT-guided PCI. Moreover, IVUS and OCT significantly reduced the risks of cardiac death and in-stent thrombosis compared to angiography. In contrast, non-conventional modalities did not alter the susceptibility to all-cause mortality, target vessel/lesion failure, myocardial infarction, revascularization, and CABG compared to conventional angiography. The evaluated data was consistent and homogenous. Our findings agree with previous meta-analyses that indicated a worse safety profile of stent implantation when performed with angiography than with IVUS or OCT [57–60].

IVUS and OCT appear to provide a safer procedure of percutaneous coronary angioplasty, likely due to the overall greater radiological performance of these modalities compared to angiography, thereby allowing more successful, more refined, and less complicated primary intervention. In particular, the examined evidence showed that IVUS is superior to angiography in terms of lower risk of MACE. IVUS permits visualizing both the coronary lumen and vessel wall at the cross-sectional level, allows characterization of the type (nature, composition, and morphology) of the plaque, and clarifies the stent failure mechanism [61, 62]. At the same time, angiography displays only the opacified luminal silhouette with minimum structural details. This limits the accurate peri-interventional assessment of the target lesion/vessel, notably exposing it to less effective and more risky stent implantation, ultimately exposing it to higher MACE incidence [61, 62].

We found that the risk of target-related revascularization was lower in patients undergoing IVUS-guided PCI than in those managed with angiography-guided PCI. Target-related revascularization is one of the standardized clinically-driven endpoints used to assess the interventional modalities’ effectiveness in coronary intervention trials [63]. It is a repeat percutaneous intervention or bypass surgery of the target lesion/vessel due to clinically significant narrowing or other complications [63]. Among the predictors of target-related revascularization are procedure- and lesion-related factors such as ostial location and use of rotablator [64]. Mainly, IVUS was found to be the advantageous modality during PCI of ostial coronary atherosclerotic plaques (i.e., aortic ostia and left anterior descending artery/left circumflex artery ostia) as such lesions prevent optimal coronary guide catheter intubation, which is required for contrast intake in both OCT and angiography [65]. Moreover, the ostium of the left main stem cannot be optimally visualized when this artery is subject to diffuse atherosclerosis. This challenge can be overcome by withdrawing the guide catheter from the left main stem, which allows for visualization of the artery’s full length. IVUS is the best modality to achieve such a maneuver [65]. Furthermore, IVUS enhanced the safety of rotational atherectomy (rotablation) [66]. Hence, due to improvements in the deliverability and cross ability of IVUS catheters, they can now be used to obtain images of the calcified lesions before and after rotational atherectomy, which would help in the selection of the appropriate guidewire and burr size, ultimately, resulting in better outcomes [66]. The unique advantages of IVUS during ostial coronary lesions and rotablation would favor lesser susceptibility to target-related revascularization.

We also observed a lower tendency to develop restenosis among patients undergoing IVUS-guided PCI. Knowing that lesion-related risk factors of coronary restenosis include lesions at the ostial location, small target vessel, lesions with complex morphology, longer stented lesions, and length of the stenosis > 20 mm [67], the observed finding can be explained by the following reasons: (i) As previously explained, IVUS can help overcome the challenges of ostial lesions, which decrease the development of restenosis. (ii) The employment of IVUS-guided PCI improved postoperative outcomes of small-vessel coronary lesions; notably prolonging event-free survival compared to angiography. That was remarkably related to coronary angiography’s higher tendency to mistakenly underestimate the real reference vessel diameters in reference to IVUS [68]. (iii) Treatment with IVUS-guided PCI was lined with a lower long-term risk of cardiac death and adverse cardiac events among patients with complex coronary artery lesions compared with angiography-guided PCI [69]. The IVUS-associated optimization of stent deployment may explain that. Thus, the IVUS-guided PCI can result in adequate stent expansion and apposition and full lesion coverage, which is due to its potential to induce larger stent size, longer stent length, higher proportion of post-dilatation, and higher inflation pressures compared to angiography-guided PCI [69]. (iv) IVUS can ameliorate the angiographic and clinical results of stent implantation for long coronary artery stenosis, as shown in the TULIP study. This study’s authors argued that IVUS motivated the operators to stent atherosclerotic segments more extensively than angiography in patients with similar stenosis lengths because of the information they received from the former modality [42]. Thus, angiography can fail to accurately identify the extent of atherosclerotic disease (underestimate it), resulting in less optimal lesion coverage. Meanwhile, IVUS defines the stenosis borders not as where significant disease begins or ends but as where compensatory vessel enlargement fails to preserve luminal dimensions [70], which would favor better stenting of large lesions and, thereby, lower restenosis likelihood.

Both IVUS and OCT reduced cardiac death in respect to angiography. Besides the interventional and imaging advantages of IVUS discussed above, OCT can produce high-resolution imaging (up to 10 μm), allowing real-time observation of the coronary structures and lesions. Thus, it can accurately measure coronary luminal parameters, identify different tissue characteristics of arterial intima and atherosclerotic plaques, and detect preoperatively vulnerable plaques and inflammation presence [71]. These would refine the immediate effect of stent implantation, which would optimize the results of the stent implantation in terms of both effectiveness and safety [71], perhaps contributing to more reduced cardiac death than conventional angiography.

Another finding is that IVUS and OCT implementation was linked with lesser risks of stent thrombosis. The latter is another event favored by lesions at small target vessels, complex lesions, those with higher lengths, or those at ostial sites or bifurcations [72]. Since IVUS can reduce the operative difficulties imposed by these lesions and allow their safer management compared to angiography (as previously discussed), it would reduce the likelihood of stent thrombosis. Likewise, it was demonstrated that PCI under OCT guidance improves clinical outcomes of patients with complex lesions and/or bifurcation lesions [21, 73, 74], which may translate to fewer stent thrombosis events.

### Study limitations

We acknowledge several limitations to the present study. First, most studies’ sample size was small, representing considerable methodological weakness. Second, patients’ selection and generalizability issues were reported in some of the included trials due to the exclusion of essential populations of patients that could benefit from PCI in real-world (e.g., those with cardiogenic shock in Wang et al. 2015 study and those with myocardial infarction in Tan et al. 2015 study). Third, the definition of our primary outcome (MACE) was heterogeneous across the RCTs and was not reported in some of them. Additionally, the limited data available for each outcome within the MACE term made them inapplicable for analysis. Finally, a large proportion of the studies used a single-center trial design, which is known to provide suboptimal data quality.

### Implications for clinical practice

In the American Heart Association 2021 guidelines, the use of IVUS and OCT during PCI has received a Class IIa recommendation, which refers to the weight of evidence/opinion in favor of usefulness/efficacy [75]. The guidelines suggest that IVUS provides useful guidance during stent implantation, particularly in cases of left main or complex lesions, allowing the prevention of ischemic events. At the same time, OCT is recommended as an alternative to IVUS except in the ostial left main disease. Our findings support these guidelines by demonstrating the clear superiority of IVUS and the relative superiority of OCT to conventional angiography. Notably, IVUS and OCT represent promising modalities for enhancing PCI efficacy and safety. Hence, the diagnostic and therapeutic advantages of IVUS/OCT should drive a shift in cardiology interventionists’ enthusiasm toward these modalities, leaving conventional angiography as the alternative instead of the standard.

Nonetheless, the non-conventional imaging techniques have many obstacles that would prevent the angiography-guided PCI era from continuing for longer than expected. One major obstacle is the accessibility issues, which would delay or even preclude the extensive generalizability of IVUS/OCT devices due to high costs and reduced availability in the market. Moreover, like any innovative procedure, interventionists’ lack of familiarity with IVUS/OCT may favor the more conventional option. However, this can be overcome through the active training of interventionists and experience sharing in scientific events and networks. Operative disadvantages also represent a key challenge that may antagonize the benefit of IVUS/OCT-guided coronary angioplasty. For instance, the currently commercialized IVUS imaging catheter has poor cross-ability for more severe stenosis or twisted angular lesions, low resolution, and suboptimal ability to assess small vascular structures [71]. Similarly, OCT increases the difficulty of PCI and limits its application in severe coronary ischemic diseases due to the necessity of blocking or removing the blood in the corresponding detection vessel [71]. These issues may be resolved with technology improvement and the acquisition of progressive expertise.

## Conclusion

In patients undergoing PCI, the current evidence shows that IVUS reduces the risks of MACE, target-vessel-related revascularization, and restenosis compared to standard angiography. However, this is not the case for OCT. Also, IVUS and OCT appear to lower the susceptibility to cardiac death and in-stent thrombosis in reference to angiography. This indicates that IVUS, followed by OCT, may be the privileged radiological technique for stent implantation whenever available. However, there is still a need for high-quality data to confirm the benefit and cost-effectiveness of these modalities in the context of coronary angioplasty.

## Electronic supplementary material

Below is the link to the electronic supplementary material.

Supplementary Material 1

## Data Availability

No datasets were generated or analysed during the current study.

## References

[1] Di Mario C, Sutaria N. Coronary angiography in the angioplasty era: projections with a meaning. Heart. 2005;91:968–76. 15958378 10.1136/hrt.2005.063107 PMC1768997

[2] Green NE, Chen S-YJ, Hansgen AR, Messenger JC, Groves BM, Carroll JD. Angiographic views used for percutaneous coronary interventions: a three-dimensional analysis of physician-determined vs. computer-generated views. Catheter Cardiovasc Interv off J Soc Card Angiogr Interv. 2005;64:451–9. 10.1002/ccd.20331 15744720

[3] Katritsis D, Webb-Peploe M. Limitations of coronary angiography: an underestimated problem? Clin Cardiol. 1991;14:20–4. 2019026 10.1002/clc.4960140106

[4] Tavakol M, Ashraf S, Brener SJ. Risks and complications of coronary angiography: a comprehensive review. Glob J Health Sci. 2012;4:65–93. 22980117 10.5539/gjhs.v4n1p65 PMC4777042

[5] Mintz GS, Guagliumi G. Intravascular imaging in coronary artery disease. Lancet (London England). 2017;390:793–809. 28831995 10.1016/S0140-6736(17)31957-8

[6] Kim Y, Bae S, Johnson TW, Son N-H, Sim DS, Hong YJ, et al. Role of Intravascular Ultrasound-guided percutaneous coronary intervention in optimizing outcomes in Acute myocardial infarction. J Am Heart Assoc. 2022;11:e023481. 35179041 10.1161/JAHA.121.023481 PMC9075077

[7] Hannan EL, Zhong Y, Reddy P, Jacobs AK, Ling FSK, King Iii SB, et al. Percutaneous coronary intervention with and without intravascular ultrasound for patients with complex lesions: utilization, mortality, and Target Vessel revascularization. Circ Cardiovasc Interv. 2022;15:e011687. 35543139 10.1161/CIRCINTERVENTIONS.121.011687

[8] Kuno T, Numasawa Y, Sawano M, Abe T, Ueda I, Kodaira M, et al. Real-world use of intravascular ultrasound in Japan: a report from contemporary multicenter PCI registry. Heart Vessels. 2019;34:1728–39. 31129872 10.1007/s00380-019-01427-9

[9] Ali ZA, Karimi Galougahi K, Mintz GS, Maehara A, Shlofmitz RA, Mattesini A. Intracoronary optical coherence tomography: state of the art and future directions. EuroIntervention J Eur Collab Work Gr Interv Cardiol Eur Soc Cardiol. 2021;17:e105–23. 10.4244/EIJ-D-21-00089 PMC9725016 34110288

[10] Olinic DM, Spinu M, Homorodean C, Ober MC, Olinic M. Real-life benefit of OCT imaging for optimizing PCI indications, Strategy, and results. J Clin Med. 2019;8. 10.3390/jcm8040437 PMC6518208 30934997

[11] Bergmark BA, Osborn EA, Ali ZA, Gupta A, Kolli KK, Prillinger JB et al. Association between Intracoronary Imaging during PCI and clinical outcomes in a real-world US Medicare Population. J Soc Cardiovasc Angiogr Interv. 2023;2. 10.1016/j.jscai.2022.100556 PMC11307420 39129806

[12] Page MJ, McKenzie JE, Bossuyt PM, Boutron I, Hoffmann TC, Mulrow CD, et al. The PRISMA 2020 statement: an updated guideline for reporting systematic reviews. Syst Rev. 2021;10:89. 33781348 10.1186/s13643-021-01626-4 PMC8008539

[13] Hutton B, Salanti G, Caldwell DM, Chaimani A, Schmid CH, Cameron C, et al. The PRISMA extension statement for reporting of systematic reviews incorporating network meta-analyses of health care interventions: checklist and explanations. Ann Intern Med. 2015;162:777–84. 26030634 10.7326/M14-2385

[14] Higgins JPTTJ, Chandler J, Cumpston M, Li T, Page MJ WV, editors. Cochrane Handbook for Systematic Reviews of Interventions. 2023.

[15] Sterne JAC, Savović J, Page MJ, Elbers RG, Blencowe NS, Boutron I, et al. RoB 2: a revised tool for assessing risk of bias in randomised trials. BMJ. 2019;366:l4898. 31462531 10.1136/bmj.l4898

[16] Ali ZA, Karimi Galougahi K, Maehara A, Shlofmitz RA, Fabbiocchi F, Guagliumi G, et al. Outcomes of optical coherence tomography compared with intravascular ultrasound and with angiography to guide coronary stent implantation: one-year results from the ILUMIEN III: OPTIMIZE PCI trial. EuroIntervention J Eur Collab Work Gr Interv Cardiol Eur Soc Cardiol. 2021;16:1085–91. 10.4244/EIJ-D-20-00498 PMC9724851 32540793

[17] Ali ZA, Landmesser U, Maehara A, Matsumura M, Shlofmitz RA, Guagliumi G, et al. Optical coherence tomography-guided versus angiography-guided PCI. N Engl J Med. 2023;389:1466–76. 37634188 10.1056/NEJMoa2305861

[18] Gaster AL, Slothuus U, Larsen J, Thayssen P, Haghfelt T. Cost-effectiveness analysis of intravascular ultrasound guided percutaneous coronary intervention versus conventional percutaneous coronary intervention. Scand Cardiovasc J. 2001;35:80–5. 11405501 10.1080/140174301750164673

[19] Gil RJ, Pawłowski T, Dudek D, Horszczaruk G, Zmudka K, Lesiak M, et al. Comparison of angiographically guided direct stenting technique with direct stenting and optimal balloon angioplasty guided with intravascular ultrasound. The multicenter, randomized trial results. Am Heart J. 2007;154:669–75. 17892989 10.1016/j.ahj.2007.06.017

[20] Habara M, Nasu K, Terashima M, Kaneda H, Yokota D, Ko E, et al. Impact of frequency-domain optical coherence tomography guidance for optimal coronary stent implantation in comparison with intravascular ultrasound guidance. Circ Cardiovasc Interv. 2012;5:193–201. 22456026 10.1161/CIRCINTERVENTIONS.111.965111

[21] Holm NR, Andreasen LN, Neghabat O, Laanmets P, Kumsars I, Bennett J, et al. OCT or Angiography Guidance for PCI in Complex Bifurcation lesions. N Engl J Med. 2023;389:1477–87. 37634149 10.1056/NEJMoa2307770

[22] Hong S-J, Kim B-K, Shin D-H, Nam C-M, Kim J-S, Ko Y-G, et al. Effect of Intravascular Ultrasound-guided vs angiography-guided Everolimus-Eluting Stent Implantation: the IVUS-XPL randomized clinical trial. JAMA. 2015;314:2155–63. 26556051 10.1001/jama.2015.15454

[23] Hong S-J, Mintz GS, Ahn C-M, Kim J-S, Kim B-K, Ko Y-G, et al. Effect of intravascular ultrasound-guided drug-eluting stent implantation: 5-Year Follow-Up of the IVUS-XPL randomized trial. JACC Cardiovasc Interv. 2020;13:62–71. 31918944 10.1016/j.jcin.2019.09.033

[24] Jakabcin J, Spacek R, Bystron M, Kvasnák M, Jager J, Veselka J, et al. Long-term health outcome and mortality evaluation after invasive coronary treatment using drug eluting stents with or without the IVUS guidance. Randomized control trial. HOME DES IVUS. Catheter Cardiovasc Interv off J Soc Card Angiogr Interv. 2010;75:578–83. 10.1002/ccd.22244 19902491

[25] Jia H, Dai J, He L, Xu Y, Shi Y, Zhao L, et al. EROSION III: a Multicenter RCT of OCT-Guided reperfusion in STEMI with Early Infarct Artery Patency. JACC Cardiovasc Interv. 2022;15:846–56. 35367176 10.1016/j.jcin.2022.01.298

[26] Kala P, Cervinka P, Jakl M, Kanovsky J, Kupec A, Spacek R, et al. OCT guidance during stent implantation in primary PCI: a randomized multicenter study with nine months of optical coherence tomography follow-up. Int J Cardiol. 2018;250:98–103. 29079414 10.1016/j.ijcard.2017.10.059

[27] Kang D-Y, Ahn J-M, Yun S-C, Hur S-H, Cho Y-K, Lee CH, et al. Optical coherence tomography-guided or intravascular ultrasound-guided percutaneous coronary intervention: the OCTIVUS Randomized Clinical Trial. Circulation. 2023;148:1195–206. 37634092 10.1161/CIRCULATIONAHA.123.066429

[28] Ali ZA, Maehara A, Généreux P, Shlofmitz RA, Fabbiocchi F, Nazif TM, et al. Optical coherence tomography compared with intravascular ultrasound and with angiography to guide coronary stent implantation (ILUMIEN III: OPTIMIZE PCI): a randomised controlled trial. Lancet (London England). 2016;388:2618–28. 27806900 10.1016/S0140-6736(16)31922-5

[29] Kim B-K, Shin D-H, Hong M-K, Park HS, Rha S-W, Mintz GS, et al. Clinical impact of intravascular ultrasound-guided chronic total occlusion intervention with Zotarolimus-Eluting Versus Biolimus-Eluting Stent Implantation: Randomized Study. Circ Cardiovasc Interv. 2015;8:e002592. 26156151 10.1161/CIRCINTERVENTIONS.115.002592

[30] Kim J-S, Kang T-S, Mintz GS, Park B-E, Shin D-H, Kim B-K, et al. Randomized comparison of clinical outcomes between intravascular ultrasound and angiography-guided drug-eluting stent implantation for long coronary artery stenoses. JACC Cardiovasc Interv. 2013;6:369–76. 23523455 10.1016/j.jcin.2012.11.009

[31] Kim J-S, Shin D-H, Kim B-K, Ko Y-G, Choi D, Jang Y, et al. Randomized comparison of stent strut coverage following angiography- or optical coherence tomography-guided percutaneous coronary intervention. Rev Esp Cardiol (Engl Ed). 2015;68:190–7. 25487222 10.1016/j.recesp.2014.07.026

[32] Kubo T, Shinke T, Okamura T, Hibi K, Nakazawa G, Morino Y, et al. Optical frequency domain imaging vs. intravascular ultrasound in percutaneous coronary intervention (OPINION trial): one-year angiographic and clinical results. Eur Heart J. 2017;38:3139–47. 29121226 10.1093/eurheartj/ehx351 PMC5837511

[33] Lee JM, Choi KH, Song Y, Bin, Lee J-Y, Lee S-J, Lee SY, et al. Intravascular imaging-guided or angiography-guided complex PCI. N Engl J Med. 2023;388:1668–79. 36876735 10.1056/NEJMoa2216607

[34] Lee S-Y, Kang D-Y, Hong S-J, Ahn J-M, Ahn C-M, Park D-W, et al. Optical coherence tomography for Coronary Bioresorbable Vascular Scaffold Implantation: a Randomized Controlled Trial. Circ Cardiovasc Interv. 2020;13:e008383. 32525410 10.1161/CIRCINTERVENTIONS.119.008383

[35] Liu XM, Yang ZM, Liu XK, Zhang Q, Liu CQ, Han Q, Le, et al. Intravascular ultrasound-guided drug-eluting stent implantation for patients with unprotected left main coronary artery lesions: a single-center randomized trial. Anatol J Cardiol. 2019;21:83–90. 30694800 10.14744/AnatolJCardiol.2018.21447 PMC6457420

[36] Mariani JJ, Guedes C, Soares P, Zalc S, Campos CM, Lopes AC, et al. Intravascular ultrasound guidance to minimize the use of iodine contrast in percutaneous coronary intervention: the MOZART (minimizing cOntrast utiliZation with IVUS Guidance in coRonary angioplasTy) randomized controlled trial. JACC Cardiovasc Interv. 2014;7:1287–93. 25326742 10.1016/j.jcin.2014.05.024 PMC4637944

[37] Mariani J, De Fazzio FR, Bernardi FLM, de Alencar Araripe Falcão B, Bezerra CG, Filho AE, et al. Minimized contrast use with intravascular ultrasound-guidance percutaneous coronary intervention. One-year follow-up of the MOZART randomized study. Rev Bras Cardiol Invasiva (English Ed. 2015;23:247–50.

[38] Meneveau N, Souteyrand G, Motreff P, Caussin C, Amabile N, Ohlmann P, et al. Optical coherence tomography to optimize results of percutaneous coronary intervention in patients with Non-ST-Elevation Acute Coronary Syndrome: results of the Multicenter, Randomized DOCTORS Study (does Optical Coherence Tomography optimize results of. Circulation. 2016;134:906–17. 27573032 10.1161/CIRCULATIONAHA.116.024393

[39] Antonsen L, Thayssen P, Maehara A, Hansen HS, Junker A, Veien KT, et al. Optical coherence tomography guided percutaneous coronary intervention with Nobori Stent Implantation in patients with Non-ST-Segment-Elevation myocardial infarction (OCTACS) trial: difference in Strut Coverage and dynamic malapposition patterns at 6 Mon. Circ Cardiovasc Interv. 2015;8:e002446. 26253735 10.1161/CIRCINTERVENTIONS.114.002446

[40] Mudra H, di Mario C, de Jaegere P, Figulla HR, Macaya C, Zahn R, et al. Randomized comparison of coronary stent implantation under ultrasound or angiographic guidance to reduce stent restenosis (OPTICUS Study). Circulation. 2001;104:1343–9. 11560848 10.1161/hc3701.096064

[41] Muramatsu T, Ozaki Y, Nanasato M, Ishikawa M, Nagasaka R, Ohota M, et al. Comparison between Optical Frequency Domain Imaging and intravascular ultrasound for percutaneous coronary intervention Guidance in Biolimus A9-Eluting stent implantation: a randomized MISTIC-1 Non-inferiority Trial. Circ Cardiovasc Interv. 2020;13:e009314. 33106049 10.1161/CIRCINTERVENTIONS.120.009314 PMC7665240

[42] Oemrawsingh PV, Mintz GS, Schalij MJ, Zwinderman AH, Jukema JW, van der Wall EE. Intravascular ultrasound guidance improves angiographic and clinical outcome of stent implantation for long coronary artery stenoses: final results of a randomized comparison with angiographic guidance (TULIP study). Circulation. 2003;107:62–7. 12515744 10.1161/01.CIR.0000043240.87526.3F

[43] Russo RJ, Silva PD, Teirstein PS, Attubato MJ, Davidson CJ, DeFranco AC, et al. A randomized controlled trial of angiography versus intravascular ultrasound-directed bare-metal coronary stent placement (the AVID Trial). Circ Cardiovasc Interv. 2009;2:113–23. 20031704 10.1161/CIRCINTERVENTIONS.108.778647

[44] Schiele F, Meneveau N, Vuillemenot A, Zhang DD, Gupta S, Mercier M, et al. Impact of intravascular ultrasound guidance in stent deployment on 6-month restenosis rate: a multicenter, randomized study comparing two strategies–with and without intravascular ultrasound guidance. RESIST Study Group. REStenosis after Ivus guided STe. J Am Coll Cardiol. 1998;32:320–8. 9708456 10.1016/S0735-1097(98)00249-6

[45] Schneider VS, Böhm F, Blum K, Riedel M, Abdelwahed YS, Klotsche J, et al. Impact of real-time angiographic co-registered optical coherence tomography on percutaneous coronary intervention: the OPTICO-integration II trial. Clin Res Cardiol. 2021;110:249–57. 32889633 10.1007/s00392-020-01739-1 PMC7862500

[46] Tan Q, Wang Q, Liu D, Zhang S, Zhang Y, Li Y. Intravascular ultrasound-guided unprotected left main coronary artery stenting in the elderly. Saudi Med J. 2015;36:549–53. 25935174 10.15537/smj.2015.5.11251 PMC4436750

[47] Tian N-L, Gami S-K, Ye F, Zhang J-J, Liu Z-Z, Lin S, et al. Angiographic and clinical comparisons of intravascular ultrasound- versus angiography-guided drug-eluting stent implantation for patients with chronic total occlusion lesions: two-year results from a randomised AIR-CTO study. EuroIntervention J Eur Collab Work Gr Interv Cardiol Eur Soc Cardiol. 2015;10:1409–17. 10.4244/EIJV10I12A245 25912391

[48] Ueki Y, Yamaji K, Barbato E, Nef H, Brugaletta S, Alfonso F, et al. Randomized comparison of Optical Coherence Tomography Versus Angiography to Guide Bioresorbable Vascular Scaffold Implantation: the OPTICO BVS Study. Cardiovasc Revasc Med. 2020;21:1244–50. 32205067 10.1016/j.carrev.2020.03.023

[49] Wang H-X, Dong P-S, Li Z-J, Wang H-L, Wang K, Liu X-Y. Application of Intravascular Ultrasound in the emergency diagnosis and treatment of patients with ST-Segment Elevation myocardial infarction. Echocardiography. 2015;32:1003–8. 25287702 10.1111/echo.12794

[50] Chamié D, Costa JRJ, Damiani LP, Siqueira D, Braga S, Costa R, et al. Optical coherence Tomography Versus Intravascular Ultrasound and Angiography to Guide Percutaneous Coronary interventions: the iSIGHT Randomized Trial. Circ Cardiovasc Interv. 2021;14:e009452. 33685212 10.1161/CIRCINTERVENTIONS.120.009452

[51] Zhang J, Gao X, Kan J, Ge Z, Han L, Lu S, et al. Intravascular Ultrasound Versus Angiography-guided drug-eluting stent implantation: the ULTIMATE Trial. J Am Coll Cardiol. 2018;72:3126–37. 30261237 10.1016/j.jacc.2018.09.013

[52] Chieffo A, Latib A, Caussin C, Presbitero P, Galli S, Menozzi A, et al. A prospective, randomized trial of intravascular-ultrasound guided compared to angiography guided stent implantation in complex coronary lesions: the AVIO trial. Am Heart J. 2013;165:65–72. 23237135 10.1016/j.ahj.2012.09.017

[53] Fallesen CO, Antonsen L, Maehara A, Noori M, Hougaard M, Hansen KN, et al. Optical coherence tomography- versus angiography-guided Magnesium Bioresorbable Scaffold Implantation in NSTEMI patients. Cardiovasc Revasc Med. 2022;40:101–10. 34949544 10.1016/j.carrev.2021.12.003

[54] Frey AW, Hodgson JM, Müller C, Bestehorn HP, Roskamm H. Ultrasound-guided strategy for provisional stenting with focal balloon combination catheter: results from the randomized strategy for Intracoronary Ultrasound-guided PTCA and Stenting (SIPS) trial. Circulation. 2000;102:2497–502. 11076823 10.1161/01.CIR.102.20.2497

[55] Gao X-F, Ge Z, Kong X-Q, Kan J, Han L, Lu S, et al. 3-Year outcomes of the ULTIMATE Trial comparing Intravascular Ultrasound Versus Angiography-guided drug-eluting stent implantation. JACC Cardiovasc Interv. 2021;14:247–57. 33541535 10.1016/j.jcin.2020.10.001

[56] Gaster AL, Slothuus Skjoldborg U, Larsen J, Korsholm L, von Birgelen C, Jensen S, et al. Continued improvement of clinical outcome and cost effectiveness following intravascular ultrasound guided PCI: insights from a prospective, randomised study. Heart. 2003;89:1043–9. 12923023 10.1136/heart.89.9.1043 PMC1767812

[57] Darmoch F, Alraies MC, Al-Khadra Y, Moussa Pacha H, Pinto DS, Osborn EA. Intravascular ultrasound imaging-guided Versus Coronary Angiography-guided percutaneous coronary intervention: a systematic review and Meta-analysis. J Am Heart Assoc. 2020;9:e013678. 32075491 10.1161/JAHA.119.013678 PMC7335557

[58] Zhang Q, Wang B, Han Y, Sun S, Lv R, Wei S. Short- and long-term prognosis of Intravascular Ultrasound-Versus Angiography-guided percutaneous coronary intervention: a Meta-analysis Involving 24,783 patients. J Interv Cardiol. 2021;2021:6082581. 34737679 10.1155/2021/6082581 PMC8536416

[59] Saylik F, Hayiroglu MI, Akbulut T, Cinar T. A comprehensive network meta-analysis: comparison of long-term outcomes between intravascular ultrasound, optical coherence tomography, and angiography-guided stent implantation. Eur Heart J. 2023;44(Supplement2):ehad655–2115.

[60] Sreenivasan J, Reddy RK, Jamil Y, Malik A, Chamie D, Howard JP, et al. Intravascular imaging–guided Versus Angiography-guided percutaneous coronary intervention: a systematic review and Meta‐analysis of Randomized trials. J Am Heart Assoc. 2024;13:e031111. 38214263 10.1161/JAHA.123.031111 PMC10926835

[61] Bourantas CV, Naka KK, Garg S, Thackray S, Papadopoulos D, Alamgir FM, et al. Clinical indications for intravascular ultrasound imaging. Echocardiography. 2010;27:1282–90. 21092059 10.1111/j.1540-8175.2010.01259.x

[62] Xu J, Lo S. Fundamentals and role of intravascular ultrasound in percutaneous coronary intervention. Cardiovasc Diagn Ther. 2020;10:1358–70. 33224762 10.21037/cdt.2020.01.15 PMC7666933

[63] Garcia-Garcia HM, McFadden EP, Farb A, Mehran R, Stone GW, Spertus J, et al. Standardized end point definitions for coronary intervention trials: the Academic Research Consortium-2 Consensus Document. Circulation. 2018;137:2635–50. 29891620 10.1161/CIRCULATIONAHA.117.029289

[64] Singh M, Gersh BJ, McClelland RL, Ho KKL, Willerson JT, Penny WF, et al. Predictive factors for ischemic target vessel revascularization in the Prevention of Restenosis with Tranilast and its outcomes (PRESTO) trial. J Am Coll Cardiol. 2005;45:198–203. 15653015 10.1016/j.jacc.2004.05.089

[65] Malaiapan Y, Leung M, White AJ. The role of intravascular ultrasound in percutaneous coronary intervention of complex coronary lesions. Cardiovasc Diagn Ther. 2020;10:1371–88. 33224763 10.21037/cdt-20-189 PMC7666921

[66] Sakakura K, Yamamoto K, Taniguchi Y, Tsurumaki Y, Momomura S-I, Fujita H. Intravascular ultrasound enhances the safety of rotational atherectomy. Cardiovasc Revasc Med. 2018;19(3 Pt A):286–91. 29113866 10.1016/j.carrev.2017.09.012

[67] Ullrich H, Olschewski M, Münzel T, Gori T. Coronary In-Stent restenosis: predictors and treatment. Dtsch Arztebl Int. 2021;118:637–44. 34379053 10.3238/arztebl.m2021.0254 PMC8715314

[68] Li L, Wang L, Zhai C-J, Mou Y-R, Wang J-H, Cui L-Q. Clinical utility of intravascular ultrasonography-guided therapy in a small-vessel coronary lesion associated with type 2 diabetes mellitus. Anatol J Cardiol. 2019;22:68–76. 31375651 10.14744/AnatolJCardiol.2019.77009 PMC6735441

[69] Choi KH, Song Y, Bin, Lee JM, Lee SY, Park TK, Yang JH, et al. Impact of Intravascular Ultrasound-guided percutaneous coronary intervention on long-term clinical outcomes in patients undergoing complex procedures. JACC Cardiovasc Interv. 2019;12:607–20. 30878474 10.1016/j.jcin.2019.01.227

[70] Escaned J, Baptista J, Di Mario C, Haase J, Ozaki Y, Linker DT, et al. Significance of automated stenosis detection during quantitative angiography. Insights gained from intracoronary ultrasound imaging. Circulation. 1996;94:966–72. 8790033 10.1161/01.CIR.94.5.966

[71] Wang J, Yuan S, Qi J, Zhang Q, Ji Z. Advantages and prospects of optical coherence tomography in interventional therapy of coronary heart disease (review). Exp Ther Med. 2022;23:255. 35261627 10.3892/etm.2022.11180 PMC8855506

[72] Gori T, Polimeni A, Indolfi C, Räber L, Adriaenssens T, Münzel T. Predictors of stent thrombosis and their implications for clinical practice. Nat Rev Cardiol. 2019;16:243–56. 30518952 10.1038/s41569-018-0118-5

[73] Burzotta F, Talarico GP, Trani C, De Maria GL, Pirozzolo G, Niccoli G, et al. Frequency-domain optical coherence tomography findings in patients with bifurcated lesions undergoing provisional stenting. Eur Hear J Cardiovasc Imaging. 2014;15:547–55. 10.1093/ehjci/jet231 24255135

[74] Chandra P, Sethuraman S, Roy S, Mohanty A, Parikh K, Charantharalyil Gopalan B, et al. Effectiveness and safety of optical coherence tomography-guided PCI in Indian patients with complex lesions: a multicenter, prospective registry. Indian Heart J. 2023;75:236–42. 37244397 10.1016/j.ihj.2023.05.008 PMC10421993

[75] Lawton JS, Tamis-Holland JE, Bangalore S, Bates ER, Beckie TM, Bischoff JM, et al. 2021 ACC/AHA/SCAI Guideline for Coronary Artery revascularization: executive summary: a report of the American College of Cardiology/American Heart Association Joint Committee on Clinical Practice guidelines. Circulation. 2022;145:e4–17. 34882436 10.1161/CIR.0000000000001039

