# Supplementary material for: Intravascular imaging-guided versus angiography-guided percutaneous coronary intervention: a systematic review and bayesian network meta-analysis of randomized controlled trials
Source: BMC Cardiovasc Disord. 2024 Sep 11;24:483. doi: 10.1186/s12872-024-04105-5 (PMC11389231; doi:10.1186/s12872-024-04105-5)
Supplement: Supplementary file 1 — Supplementary Material 1 [file 12872_2024_4105_MOESM1_ESM.docx]

**Supplementary material:**

**Title.**

**Intravascular Imaging-Guided versus Angiography-Guided Percutaneous Coronary Intervention: A Systematic Review and Bayesian Network Meta-analysis of Randomized Controlled Trials.**

**Running Title.**

Intravascular Imaging-Guided versus Angiography-Guided PCI

**Authors.**

Ahmed Mazen Amin^1^, Yehya Khlidj^2^, Mohamed Abuelazm^3^, Ahmed Sayed^4^, Ubaid Khan^5^, Mariam Mahmoud Elewidi^3^, Mohammad Tanashat^6^, Hesham Elharti^3^, Mohamed Ellabban^7^, Abdullah K. Alassiri^8^, Mohamad Alsaed^9^, Basel Abdelazeem^10^, Akram Kawsara^10^_._

**Affiliations.**

1. Faculty of Medicine, Mansoura University, Mansoura, Egypt.
2. Faculty of Medicine, Algiers University, Algiers, Algeria.
3. Faculty of Medicine, Tanta University, Tanta, Egypt.
4. Faculty of Medicine, Ain Shams University, Cairo, Egypt.
5. Division of Cardiology, University of Maryland, School of Medicine, Baltimore, USA.
6. Faculty of Medicine, Yarmouk University, Irbid, Jordan.
7. Faculty of Medicine, Al-Azhar University, Cairo, Egypt.
8. Faculty of Medicine, King Abdulaziz University, Jeddah, Saudi Arabia.
9. Department of Medicine, West Virginia University, Morgantown, West Virginia, USA.
10. Department of Cardiology, West Virginia University, Morgantown, West Virginia, USA.

**Keywords.**

Intravascular ultrasound, optical coherence tomography, angiography, coronary artery disease, percutaneous coronary intervention.

**Contents:**

**Tables.**Table S1: Search strategy.

Table S2: Assessments of inconsistency and heterogeneity.

**Figures.**Figure S1: Forest plot of MACE (fixed effects model).

Figure S2: Forest plot of MACE (random effects model).

Figure S3: Forest plot of all-cause mortality (fixed effects model).

Figure S4: Forest plot of all-cause mortality (random effects model).

Figure S5: Forest plot of cardiac death (fixed effects model).

Figure S6: Forest plot of cardiac death (random effects model).

Figure S7: Forest plot of target vessel failure (fixed effects model).

Figure S8: Forest plot of target vessel failure (random effects model).

Figure S9: Forest plot of target lesion failure (fixed effects model).

Figure S10: Forest plot of target lesion failure (random effects model).

Figure S11: Forest plot of myocardial infarction (fixed effects model).

Figure S12: Forest plot of myocardial infarction (random effects model).

Figure S13: Forest plot of any revascularization (fixed effects model).

Figure S14: Forest plot of any revascularization (random effects model).

Figure S15: Forest plot of target-vessel-related revascularization (fixed effects model).

Figure S16: Forest plot of target-vessel-related revascularization (random effects model).

Figure S17: Forest plot of CABG (fixed effects model).

Figure S18: Forest plot of CABG (random effects model).

Figure S19: Forest plot of stent thrombosis (fixed effects model).

Figure S20: Forest plot of stent thrombosis (random effects model).

Figure S21: Forest plot of re-stenosis (fixed effects model).

Figure S22: Forest plot of re-stenosis (random effects model).

Figure S23: Funnel plot of MACE.

Figure S24: Funnel plot of all-cause mortality.

Figure S25: Funnel plot of cardiac death.

Figure S26: Funnel plot of target-vessel failure.

Figure S27: Funnel plot of target-lesion failure.

Figure S28: Funnel plot of myocardial infarction.

Figure S29: Funnel plot of any revascularization.

Figure S30: Funnel plot of target-vessel revascularization.

Figure S31: Funnel plot of CABG.

Figure S32: Funnel plot of stent thrombosis.

Figure S33: Funnel plot of re-stenosis.

| Database | Search Terms | Search Field | Search Results |
| --- | --- | --- | --- |
| Pubmed | ("intravascular ultrasound" OR IVUS OR "optical coherence tomography" OR "optical frequency domain" OR OCT) AND ("percutaneous coronary intervention" OR PCI OR "stent implantation" OR "stent placement" OR "DES-implantation") Filters: Clinical Trial | All Field | 1570 |
| Cochrane | ("intravascular ultrasound" OR IVUS OR "optical coherence tomography" OR "optical frequency domain" OR OCT) AND ("percutaneous coronary intervention" OR PCI OR "stent implantation" OR "stent placement" OR "DES-implantation") | All Field | 1358 |
| WOS | ("intravascular ultrasound" OR IVUS OR "optical coherence tomography" OR "optical frequency domain" OR OCT) AND ("percutaneous coronary intervention" OR PCI OR "stent implantation" OR "stent placement" OR "DES-implantation") AND trial | All Field | 1410 |
| SCOPUS | ("intravascular ultrasound" OR IVUS OR "optical coherence tomography" OR "optical frequency domain" OR OCT) AND ("percutaneous coronary intervention" OR PCI OR "stent implantation" OR "stent placement" OR "DES-implantation") AND trial | Title, Abstract, Keywords | 1569 |
| EMBASE | #4.  #1 AND #2 AND #3                                           504  #3.  trials:ti,ab,kw                                      1,011,175  #2.  'percutaneous coronary intervention':ti,ab,kw OR       136,296       pci:ti,ab,kw OR 'stent implantation':ti,ab,kw OR       'stent placement':ti,ab,kw OR 'des       implantation':ti,ab,kw  #1.  'intravascular ultrasound':ti,ab,kw OR                 122,430       ivus:ti,ab,kw OR 'optical coherence       tomography':ti,ab,kw OR 'optical frequency       domain':ti,ab,kw OR oct:ti,ab,kw  ....................................................... | All Field | 504 |

**Table S1: Search Strategy**

| **Outcome** | **Direct estimate of IVUS versus OCT (if available)*** | **Indirect estimate of IVUS versus OCT (if available)*** | **P-value for inconsistency between direct and indirect estimates*** | **N of comparisons between conventional angiography and intravascular ultrasonography** | **N of comparisons between conventional angiography and optical coherence tomography** | **N of comparisons between intravascular ultrasonography and optical coherence tomography** | **Heterogeneity of comparisons between conventional angiography and intravascular ultrasonography (I-squared; %)†** | **Heterogeneity of comparisons between conventional angiography and optical coherence tomography (I-squared; %)†** | **Heterogeneity of comparisons between intravascular ultrasonography and optical coherence tomography (I-squared; %)†** |
| --- | --- | --- | --- | --- | --- | --- | --- | --- | --- |
| MACE | 0.94 (0.45 to 1.97) | 1.05 (0.64 to 1.74) | 0.81 | 16 | 7 | 2 | 24.57 | 0.00 | 0.00 |
| All-cause mortality | 0.43 (0.20 to 0.89) | 0.75 (0.38 to 1.32) | 0.26 | 15 | 9 | 4 | 0.00 | 0.00 | 0.00 |
| Cardiac death | 0.29 (0.11 to 0.80) | 1.30 (0.66 to 2.91) | 0.01 | 14 | 7 | 3 | 0.00 | 0.00 | 0.00 |
| Target vessel failure | 0.71 (0.36 to 1.44) | 1.48 (0.57 to 3.56) | 0.15 | 3 | 2 | 2 | 0.00 | 0.00 | 0.00 |
| Target lesion failure | 0.60 (0.25 to 1.62) | 1.49 (0.29 to 5.65) | 0.25 | 2 | 2 | 2 | 20.88 | 0.00 | 0.00 |
| Myocardial infarction | 0.54 (0.25 to 1.19) | 1.28 (0.58 to 2.80) | 0.12 | 17 | 8 | 4 | 31.68 | 0.00 | 0.00 |
| Revascularization | 0.72 (0.38 to 1.28) | 1.23 (0.67 to 2.31) | 0.18 | 6 | 4 | 3 | 7.95 | 0.00 | 0.00 |
| Target-related revascularization | 0.50 (0.25 to 0.93) | 1.57 (1.04 to 2.26) | 0.003 | 15 | 8 | 3 | 0.00 | 0.00 | 0.00 |
| Stent thrombosis | 0.21 (0.06 to 0.67) | 1.25 (0.46 to 2.89) | 0.03 | 14 | 9 | 3 | 0.00 | 0.00 | 0.00 |
| CABG | 0.47 (0.10 to 2.59) | 0.86 (0.15 to 6.63) | 0.61 | 7 | 1 | 1 | 46.42 | Not applicable | Not applicable |
| Restenosis | Not applicable | Not applicable | Not applicable | 8 | 2 | 0 | 37.74 | 0.00 | Not applicable |
| *Node-splitting could not be performed for outcomes with an absent pairwise comparison or which were based on only 2 trials. | | | | | | | | | |
| †Heterogeneity could not be assessed for outcomes with zero or 1 comparisons. | | | | | | | | | |

***Table S2: Assessments of inconsistency and heterogeneity***


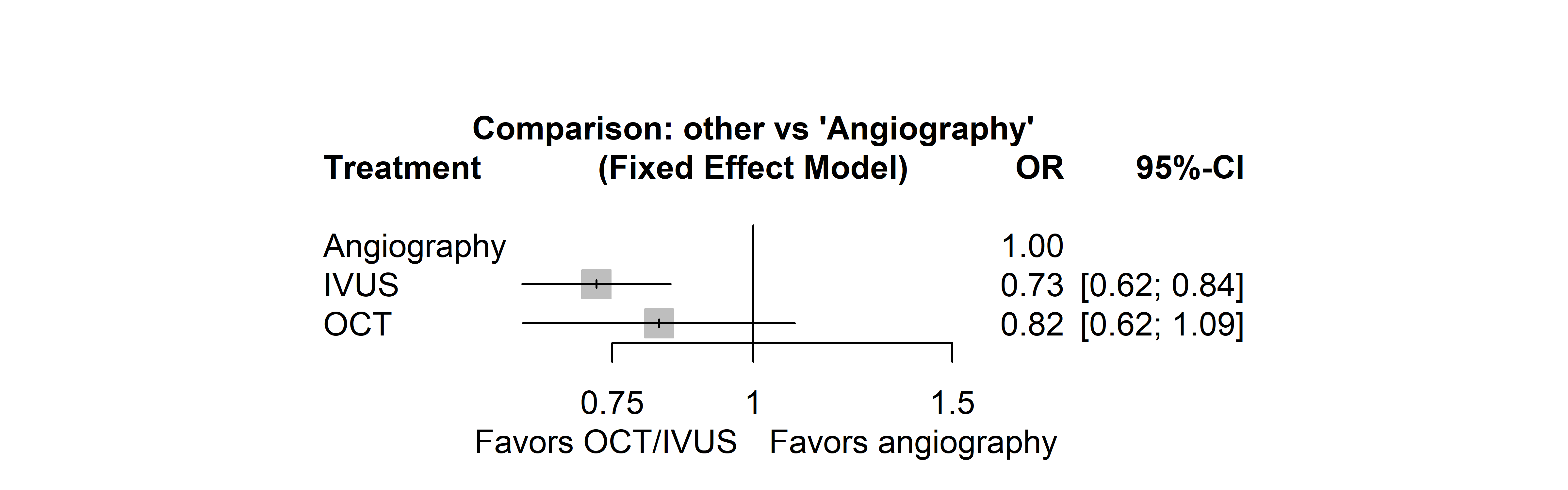


***Figure S1: Forest plot of MACE (fixed effects model).***

***
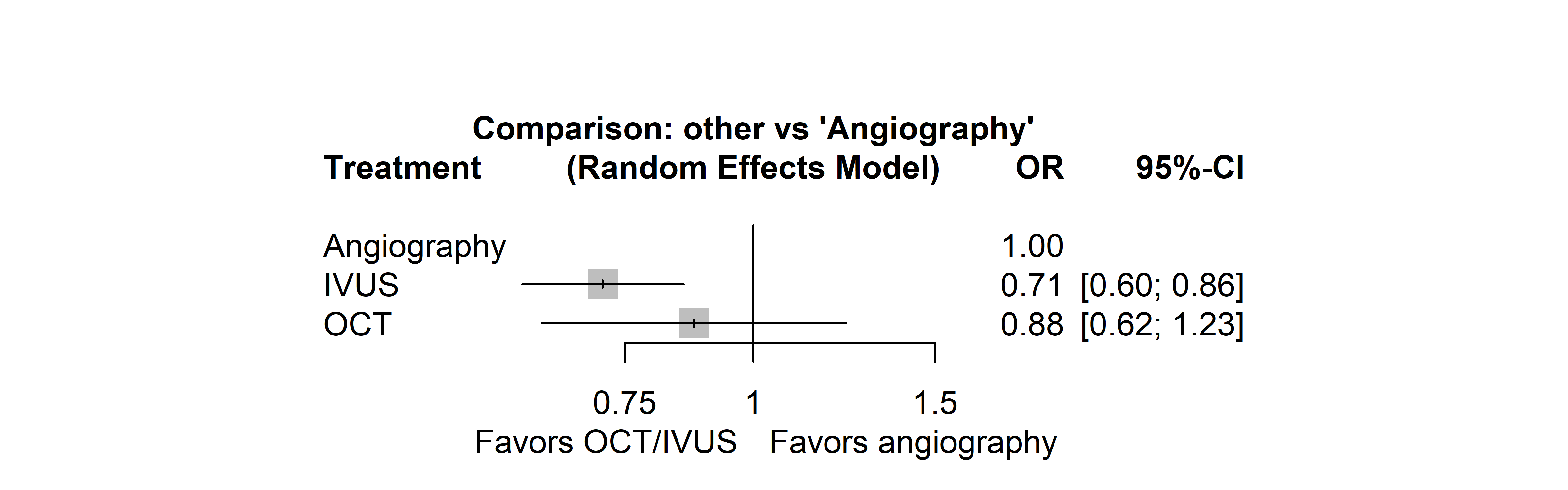
***

***Figure S2: Forest plot of MACE (random effects model).***

***
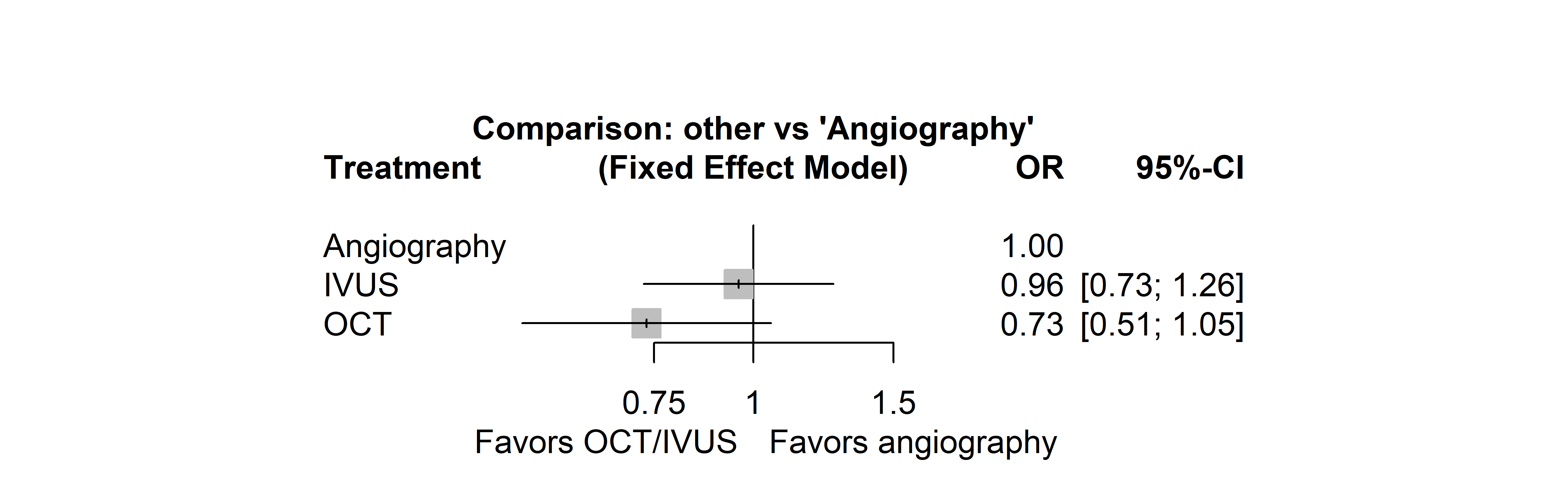
***

***Figure S3: Forest plot of all-cause mortality (fixed effects model).***

***
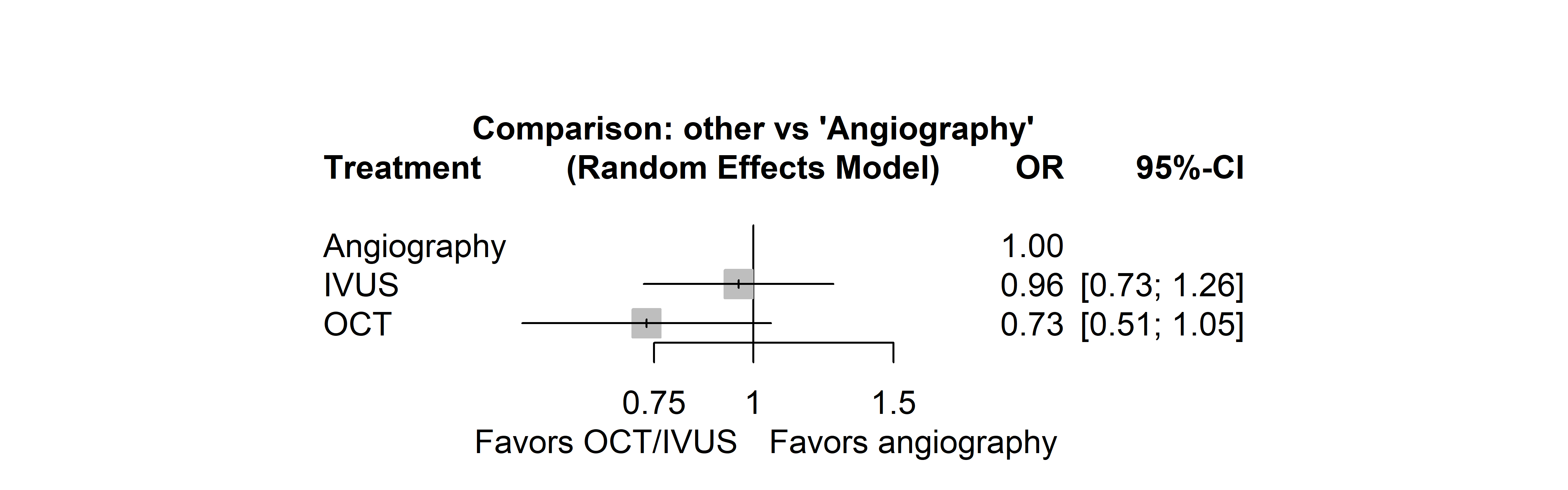
***

***Figure S4: Forest plot of all-cause mortality (random effects model).***

***
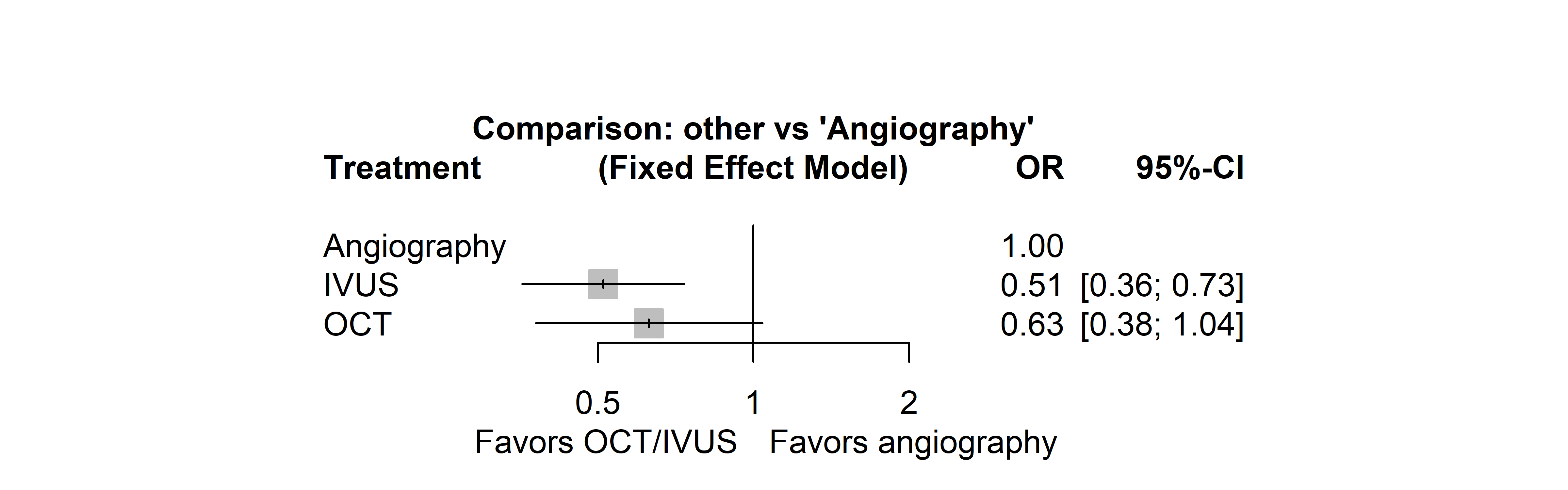
***

***Figure S5: Forest plot of cardiac death (fixed effects model).***

***
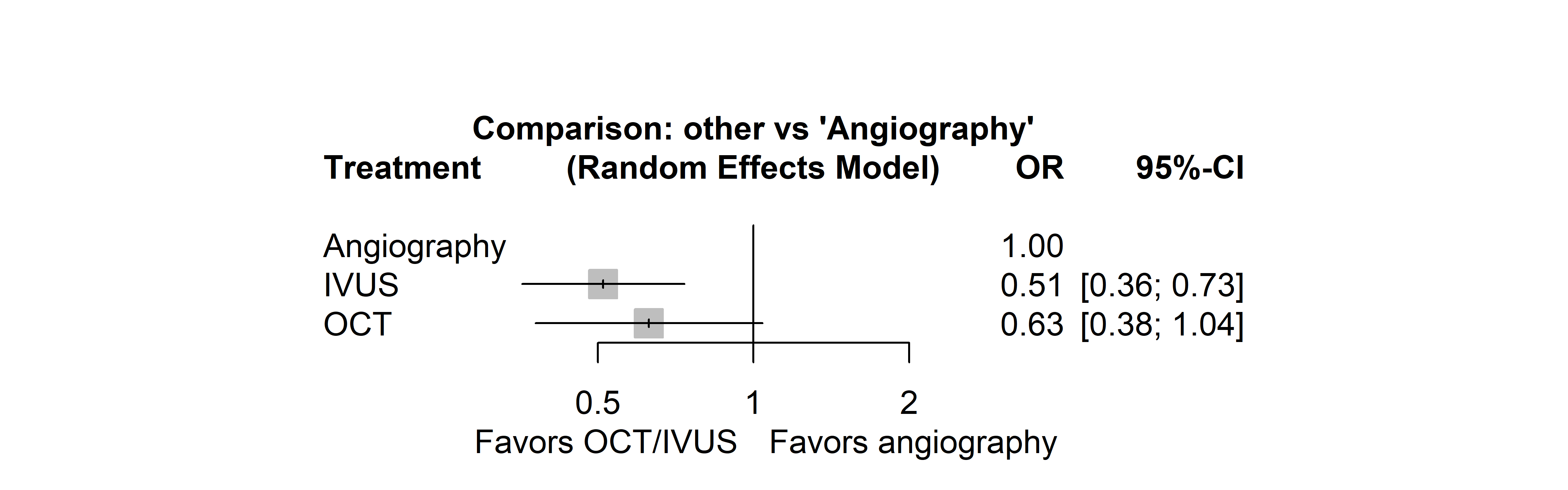
***

***Figure S6: Forest plot of cardiac death (random effects model).***

***
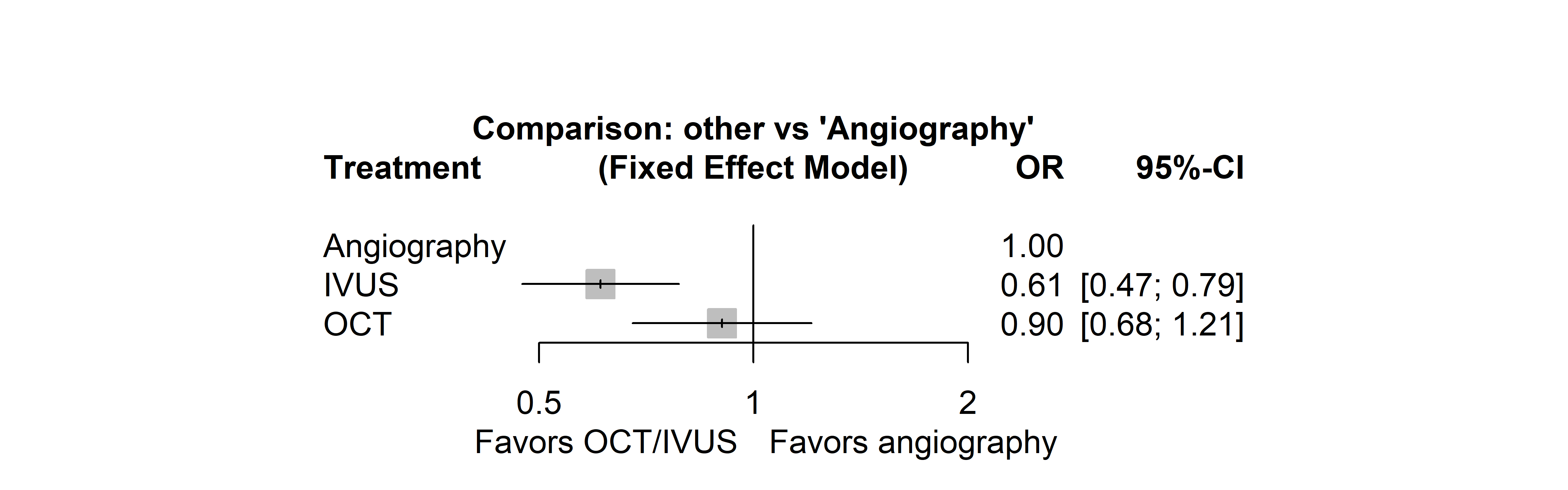
***

***Figure S7: Forest plot of target vessel failure (fixed effects model).***

***
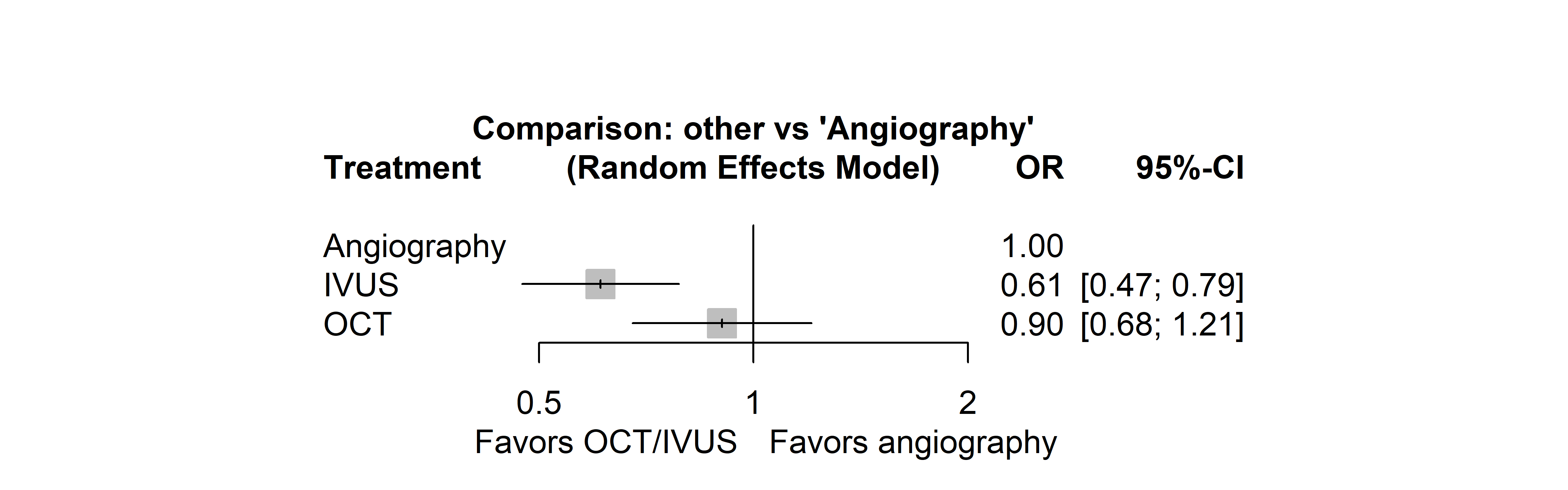
***

***Figure S8: Forest plot of target vessel failure (random effects model).***

***
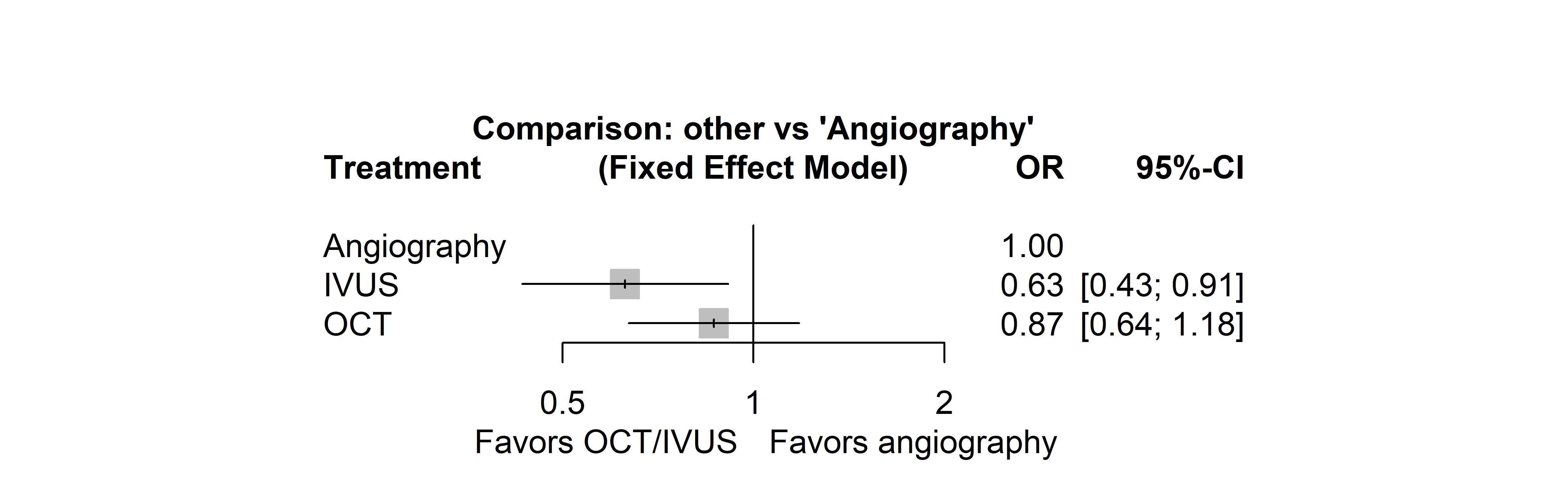
***

***Figure S9: Forest plot of target lesion failure (fixed effects model).***

***
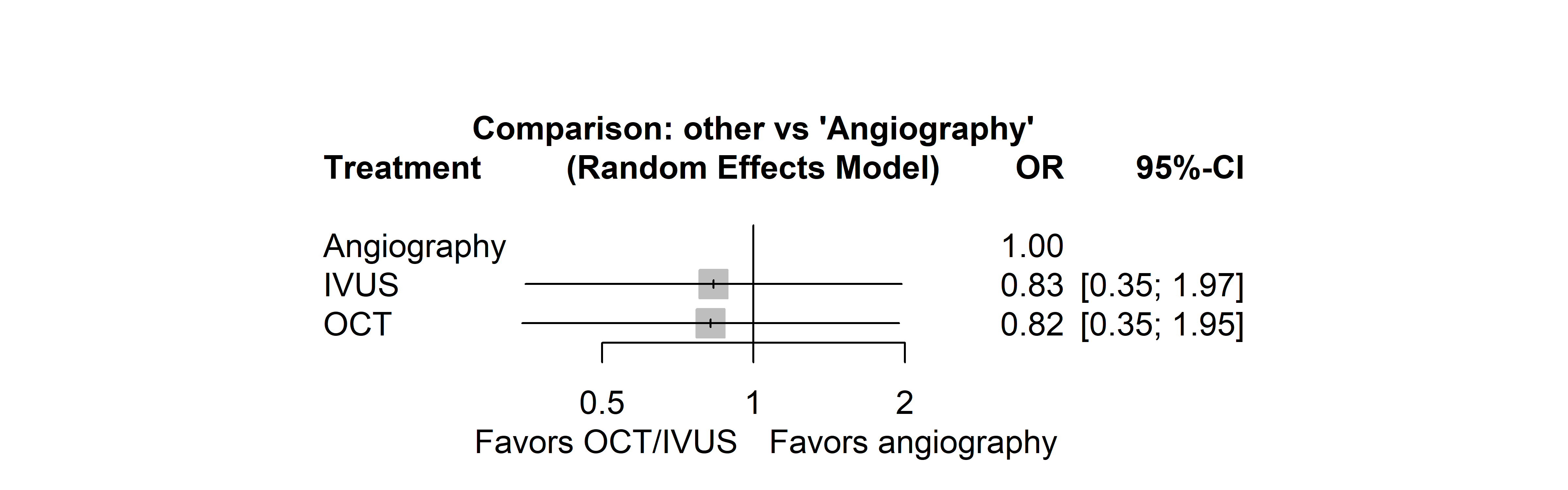
***

***Figure S10: Forest plot of target lesion failure (random effects model).***

***
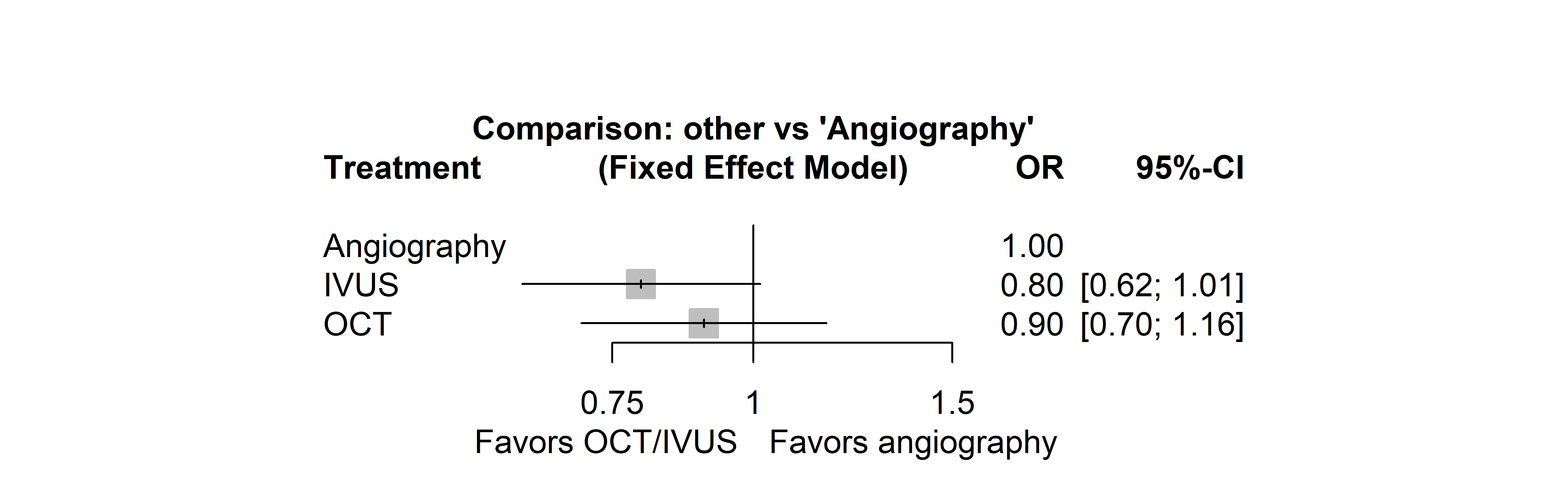
***

***Figure S11: Forest plot of myocardial infarction (fixed effects model).***

***
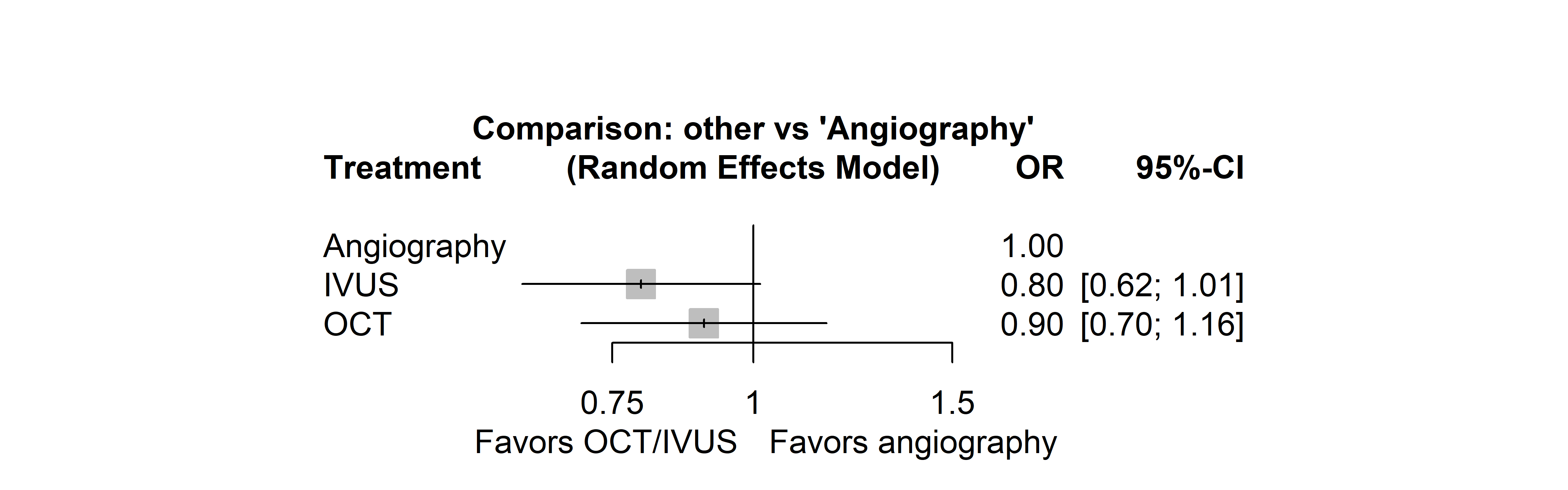
***

***Figure S12: Forest plot of myocardial infarction (random effects model).***

***
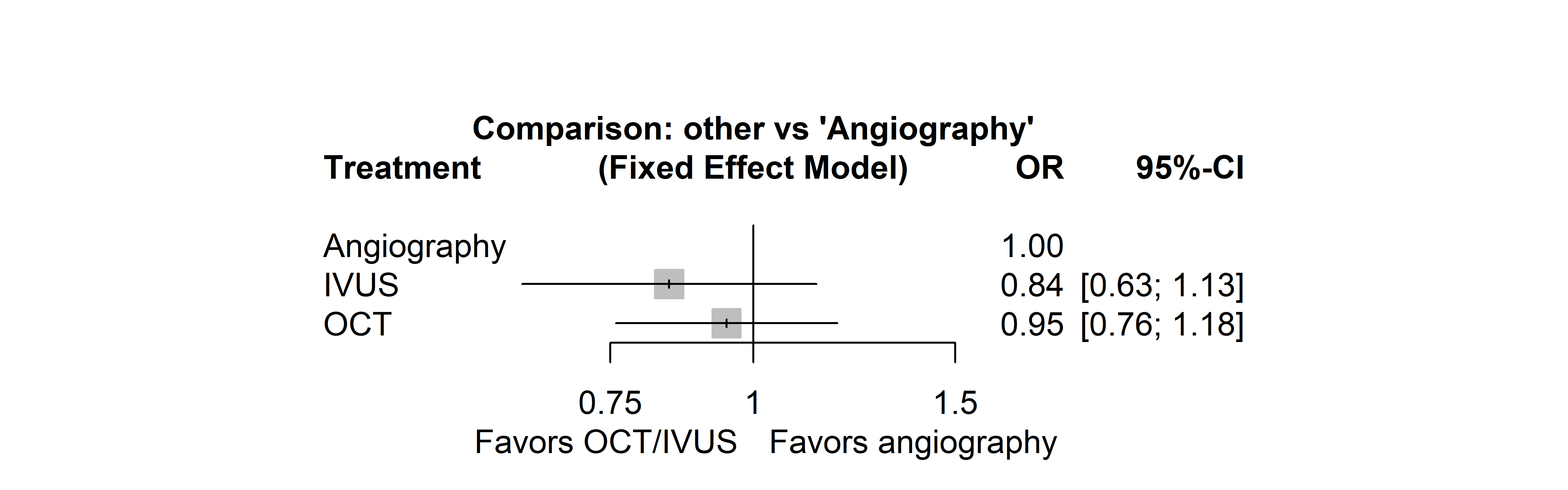
***

***Figure S13: Forest plot of any revascularization (fixed effects model).***

***
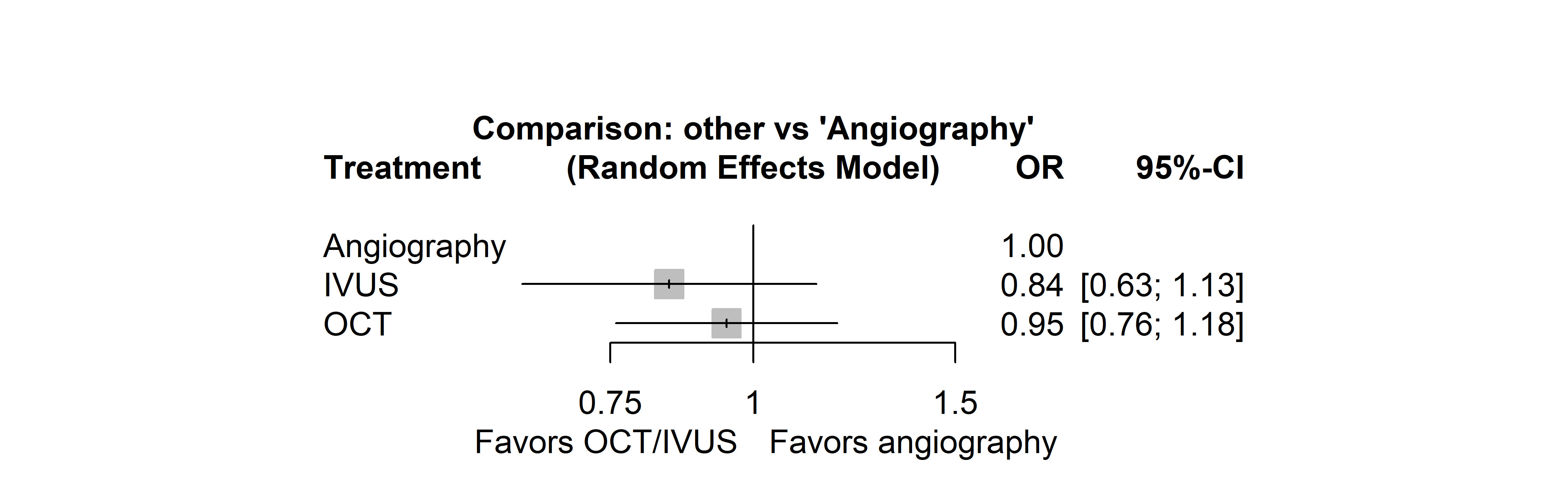
***

***Figure S14: Forest plot of any revascularization (random effects model).***

***
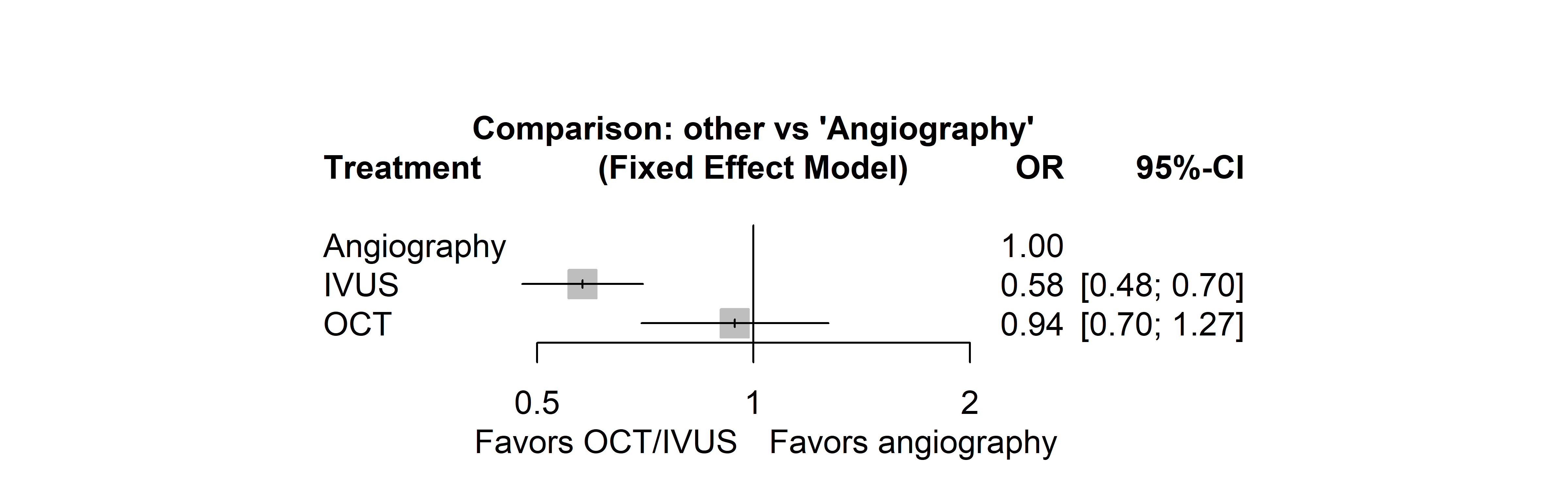
***

***Figure S15: Forest plot of target-vessel-related revascularization (fixed effects model).***

***
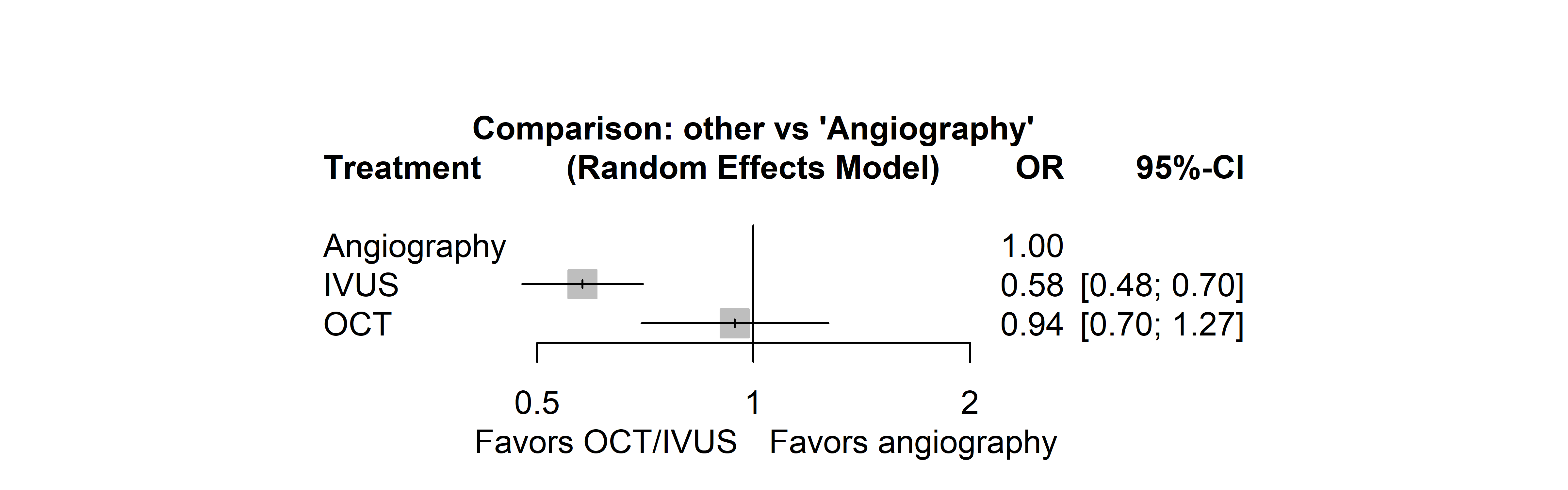
***

***Figure S16: Forest plot of target-vessel-related revascularization (random effects model).***

***
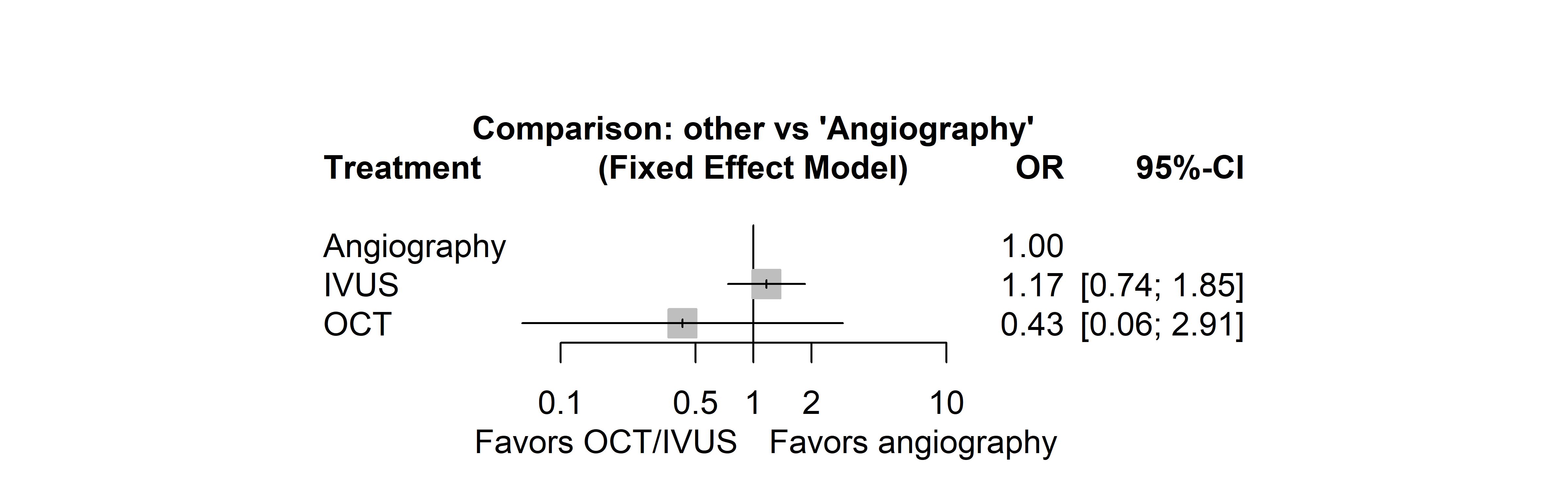
***

***Figure S17: Forest plot of CABG (fixed effects model).***

***
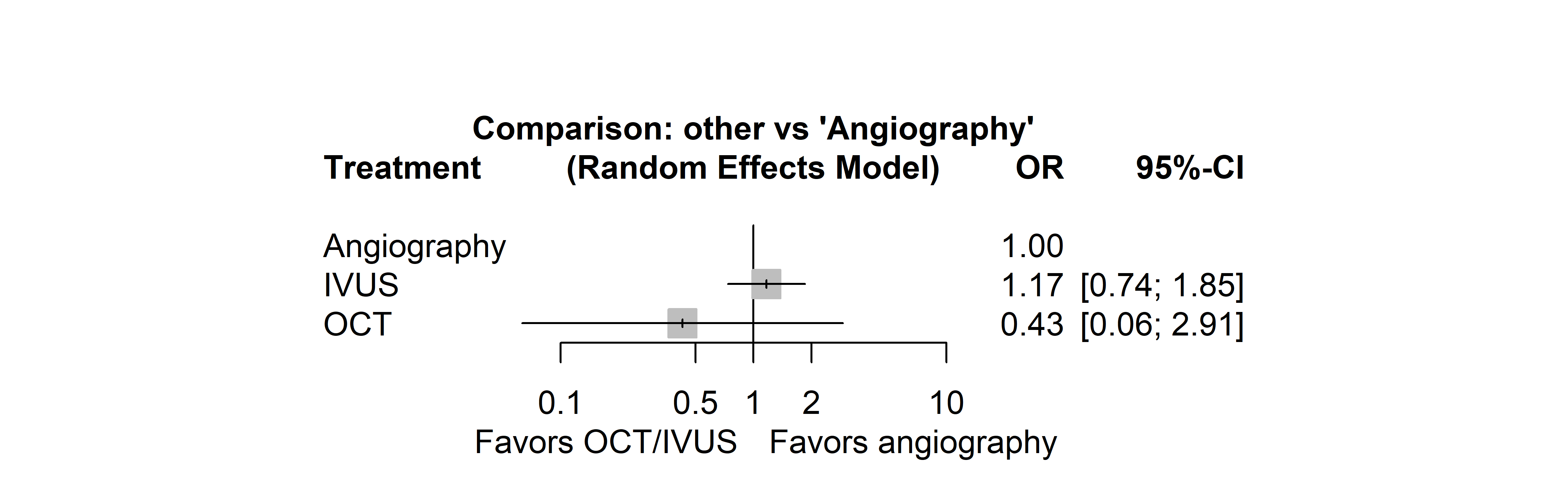
***

***Figure S18: Forest plot of CABG (random effects model).***

***
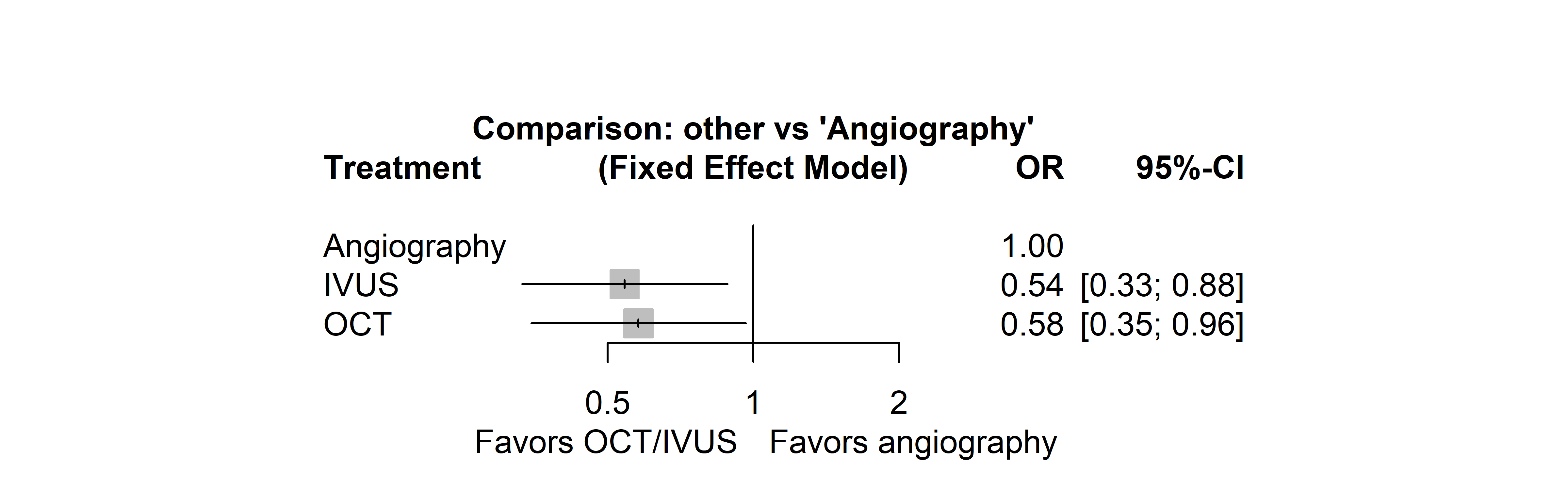
***

***Figure S19: Forest plot of stent thrombosis (fixed effects model).***

***
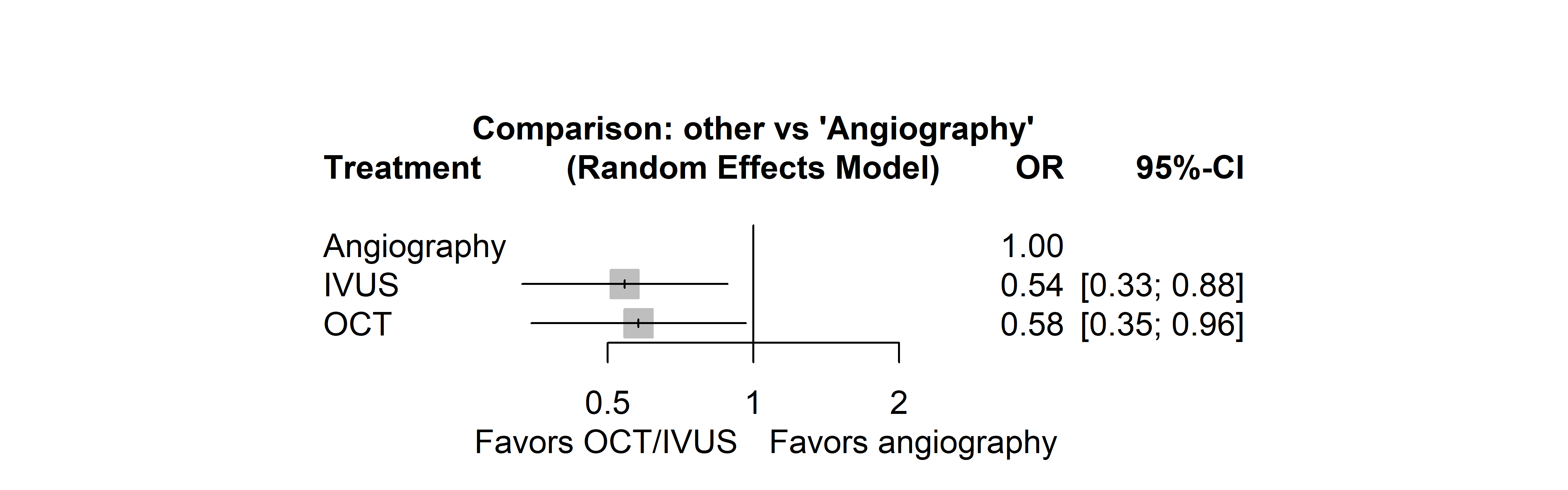
***

***Figure S20: Forest plot of stent thrombosis (random effects model).***

***
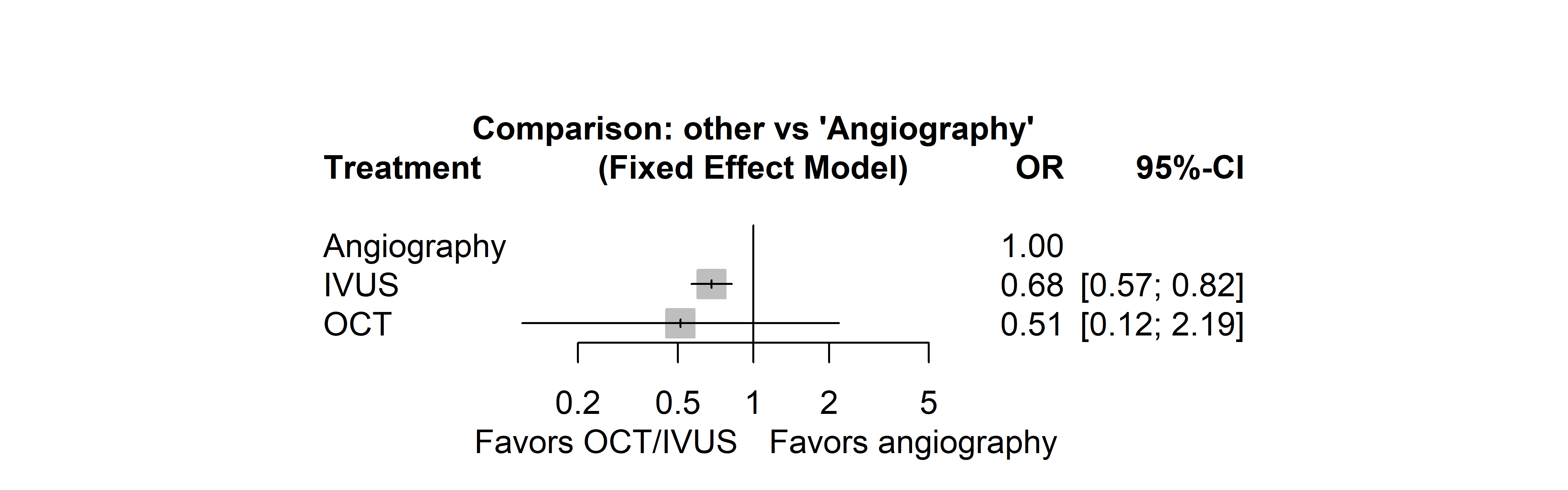
***

***Figure S21: Forest plot of re-stenosis (fixed effects model).***

***
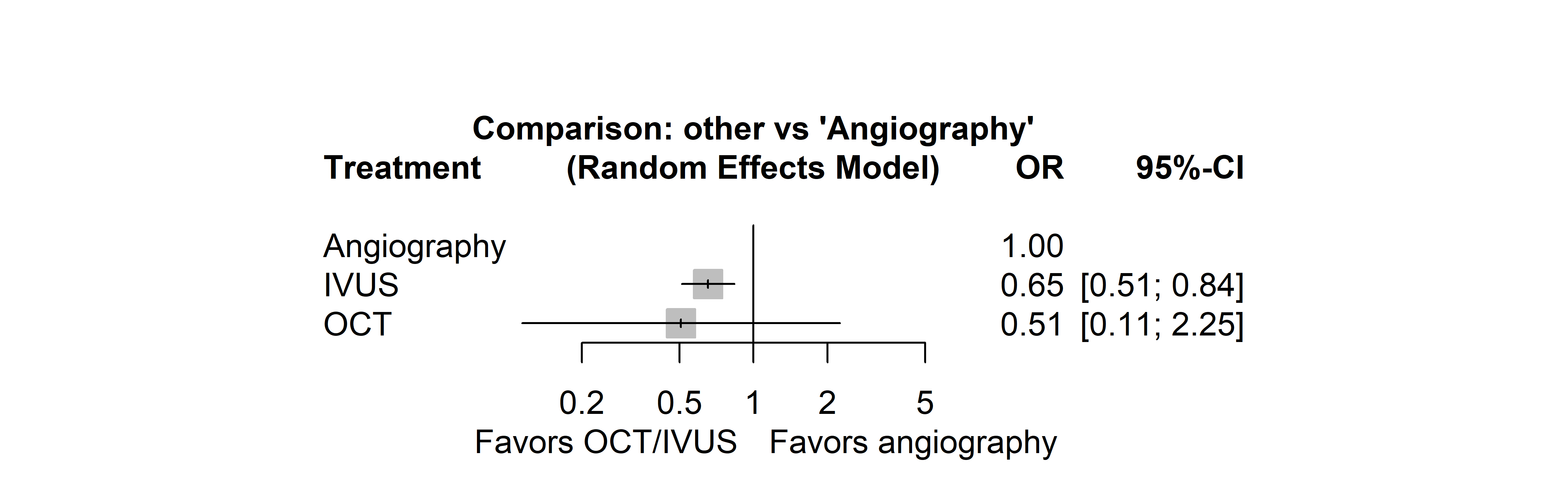
***

***Figure S22: Forest plot of re-stenosis (random effects model).***

***
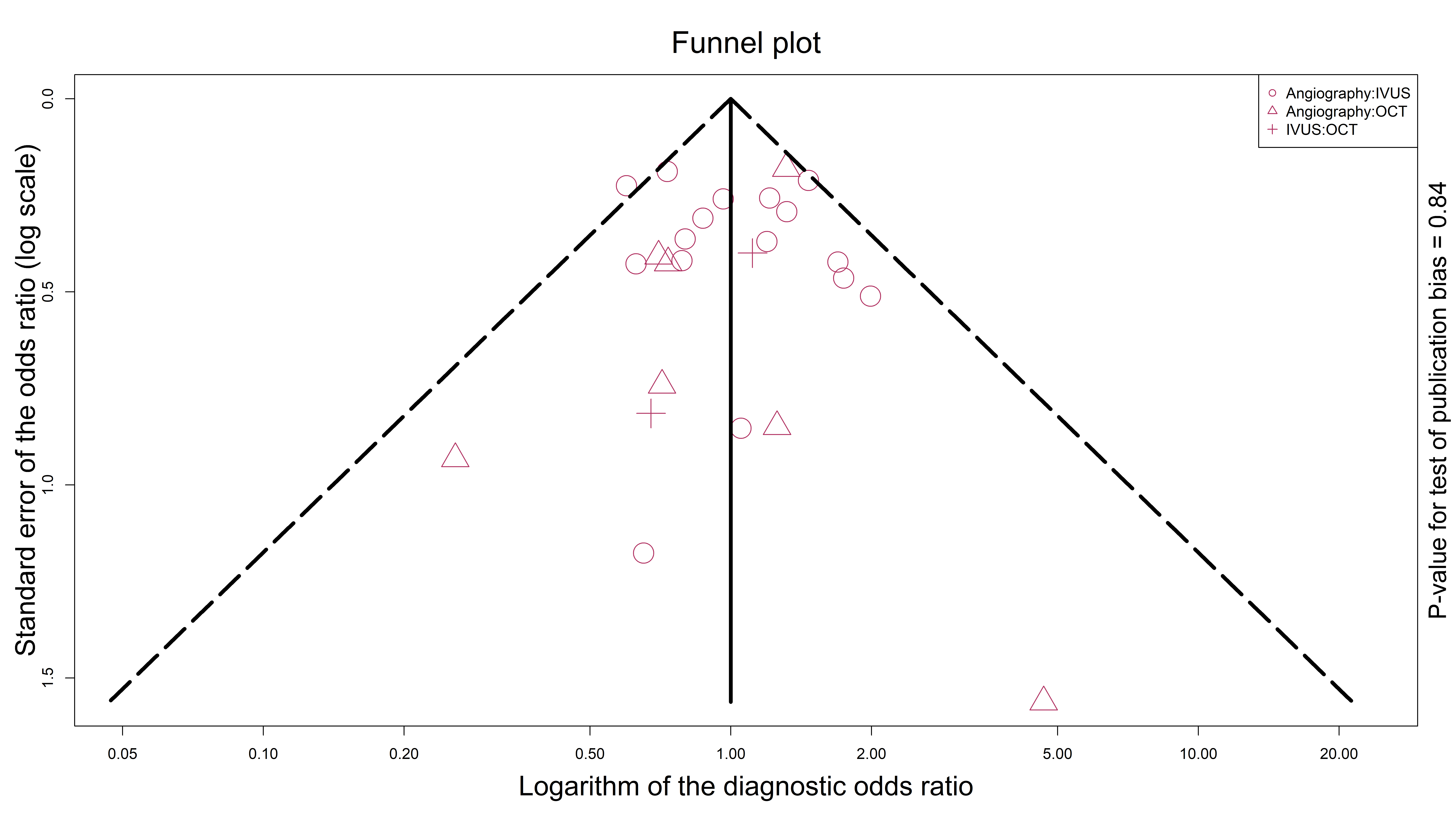
***

***Figure S23: Funnel plot of MACE.***

***
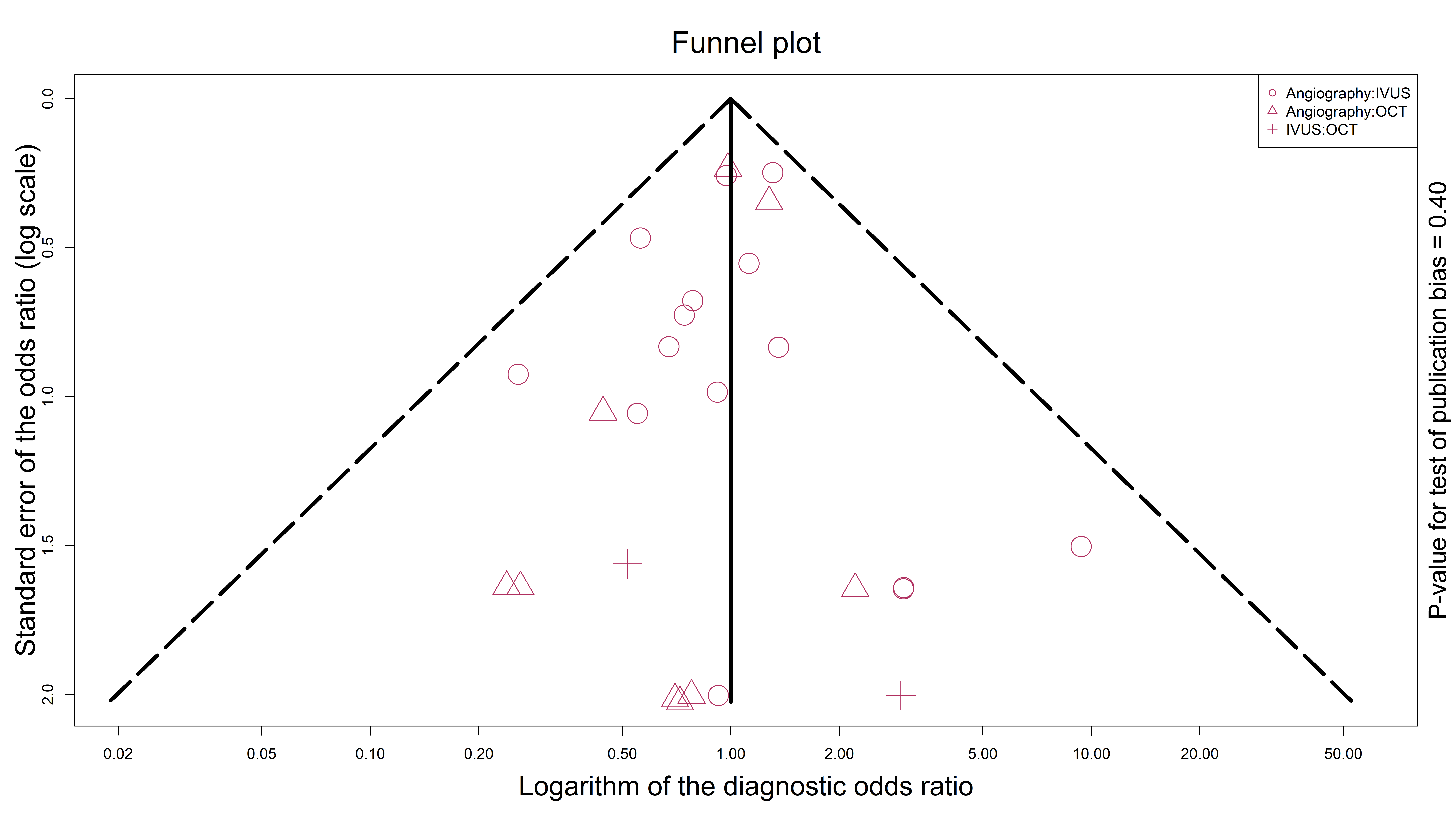
***

***Figure S24: Funnel plot of all-cause mortality.***

***
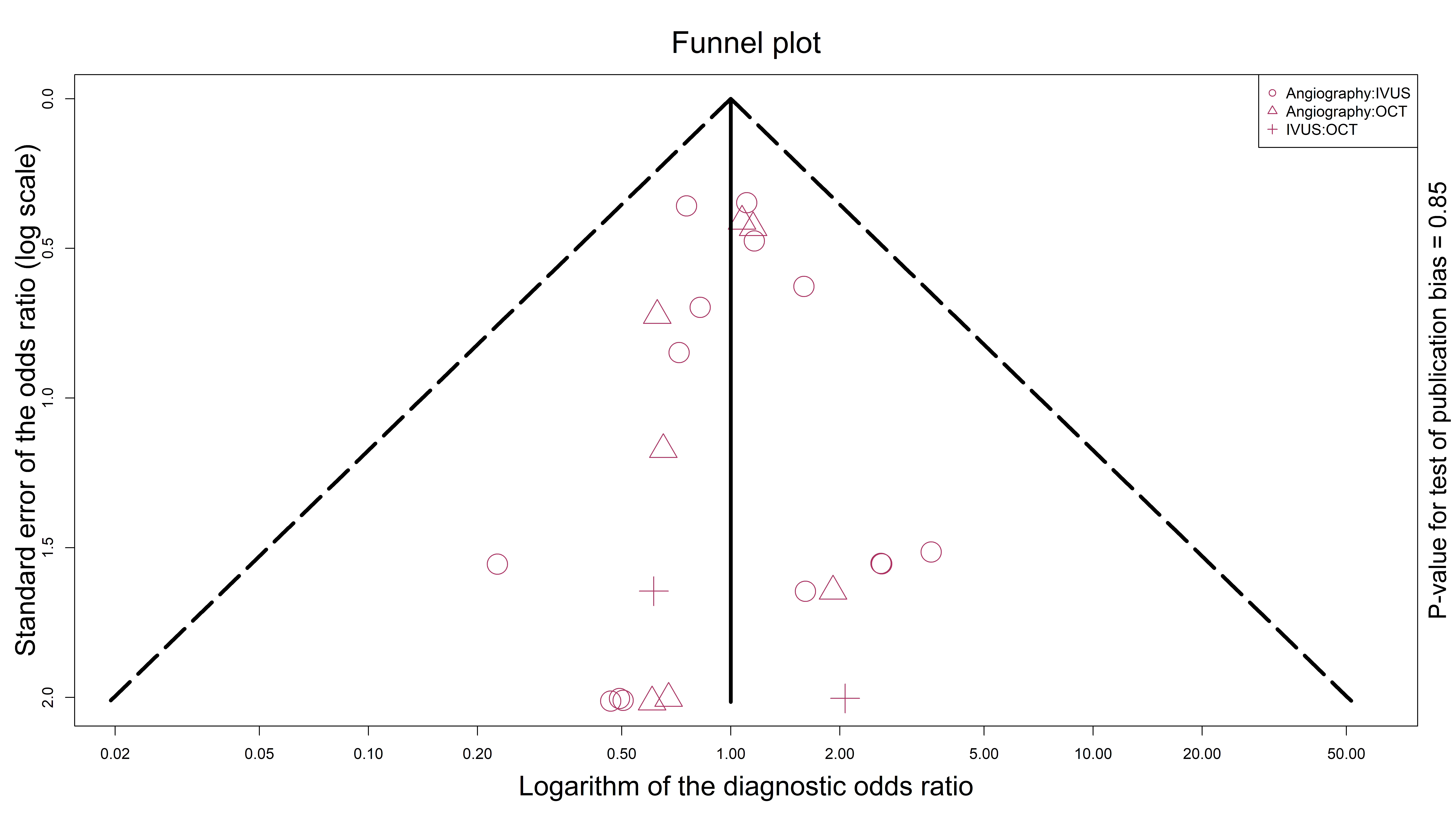
***

***Figure S25: Funnel plot of cardiac death.***

***
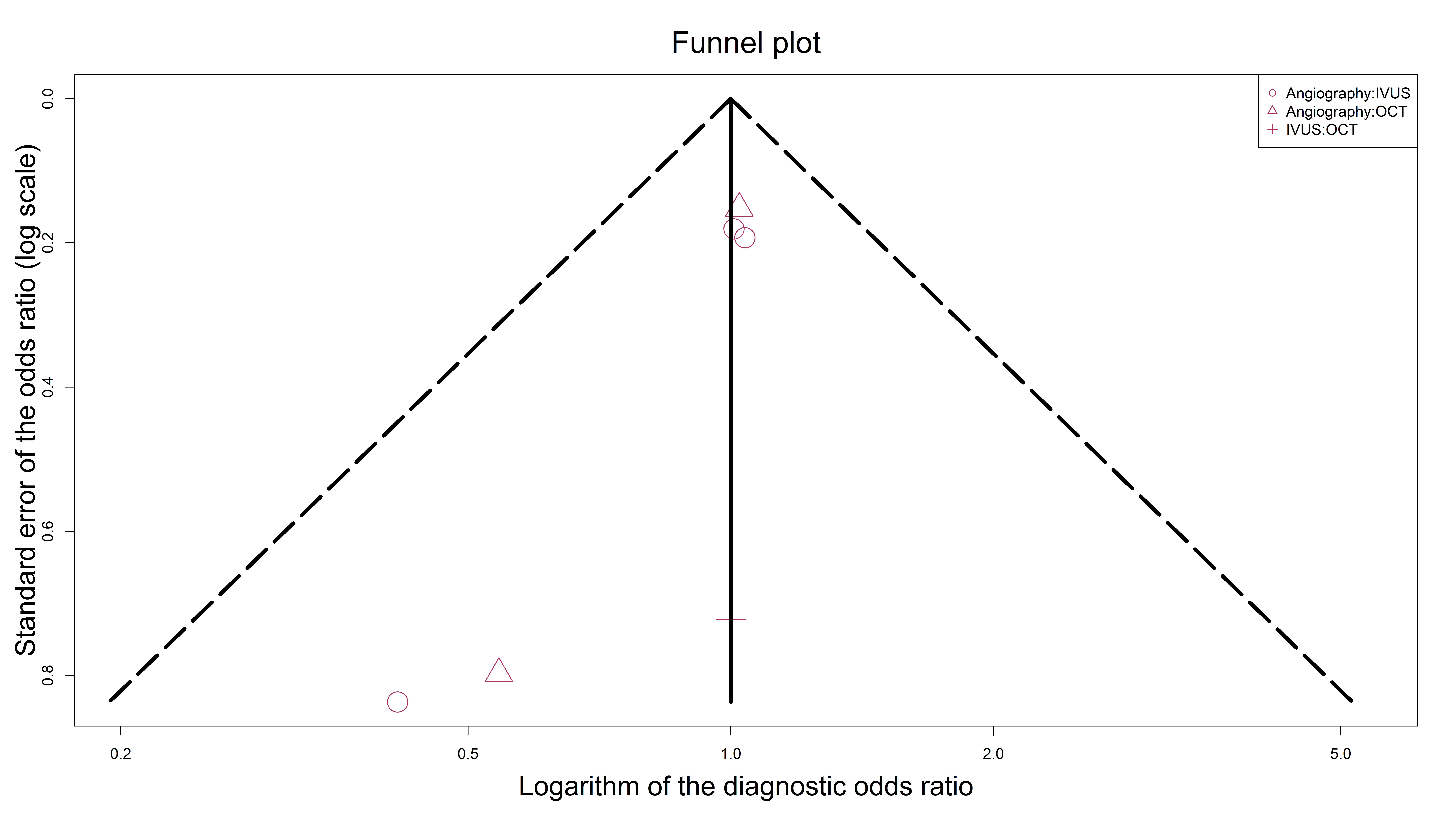
***

***Figure S26: Funnel plot of target vessel failure.***

***
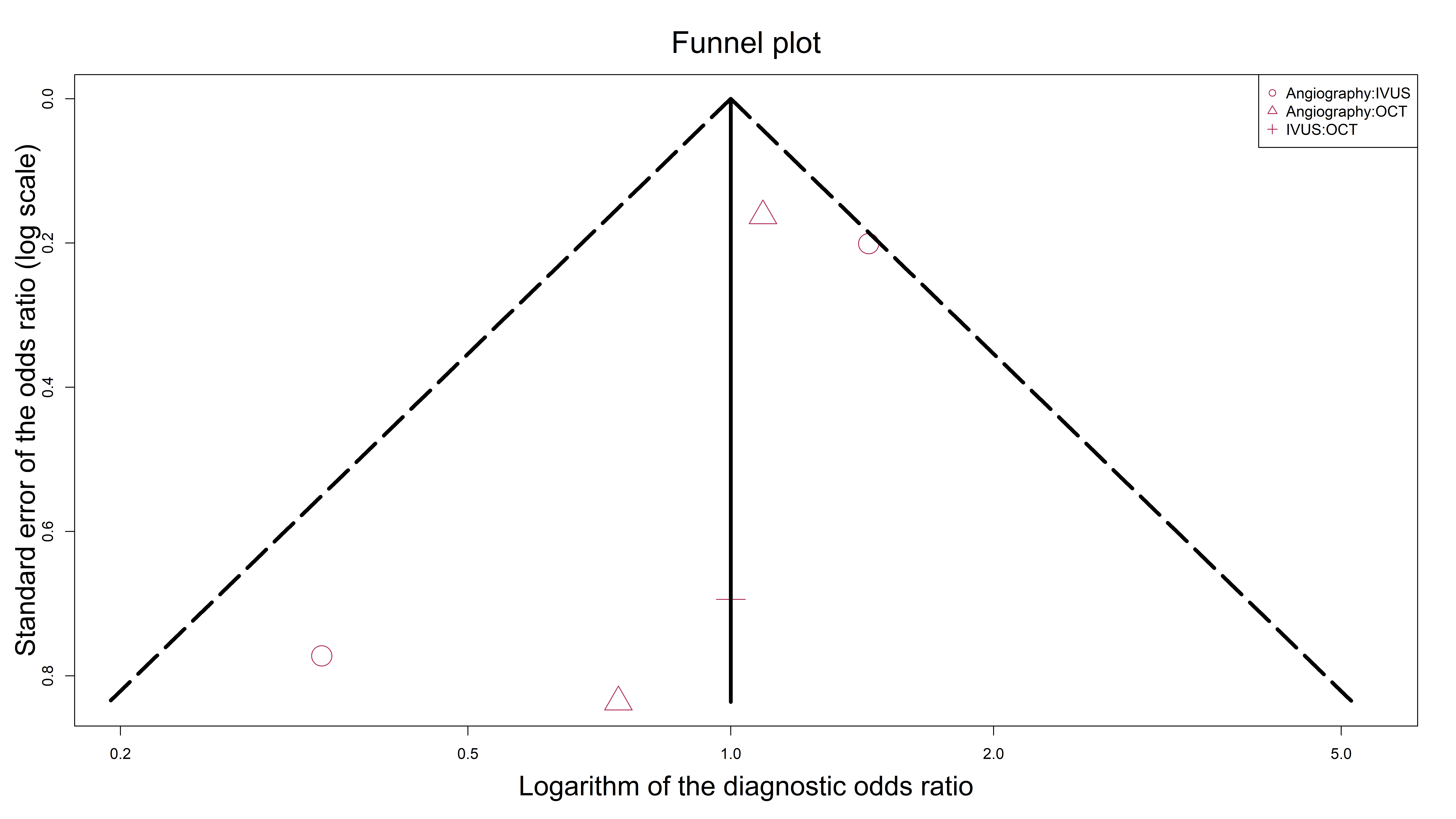
***

***Figure S27: Funnel plot of target-lesion failure.***

***
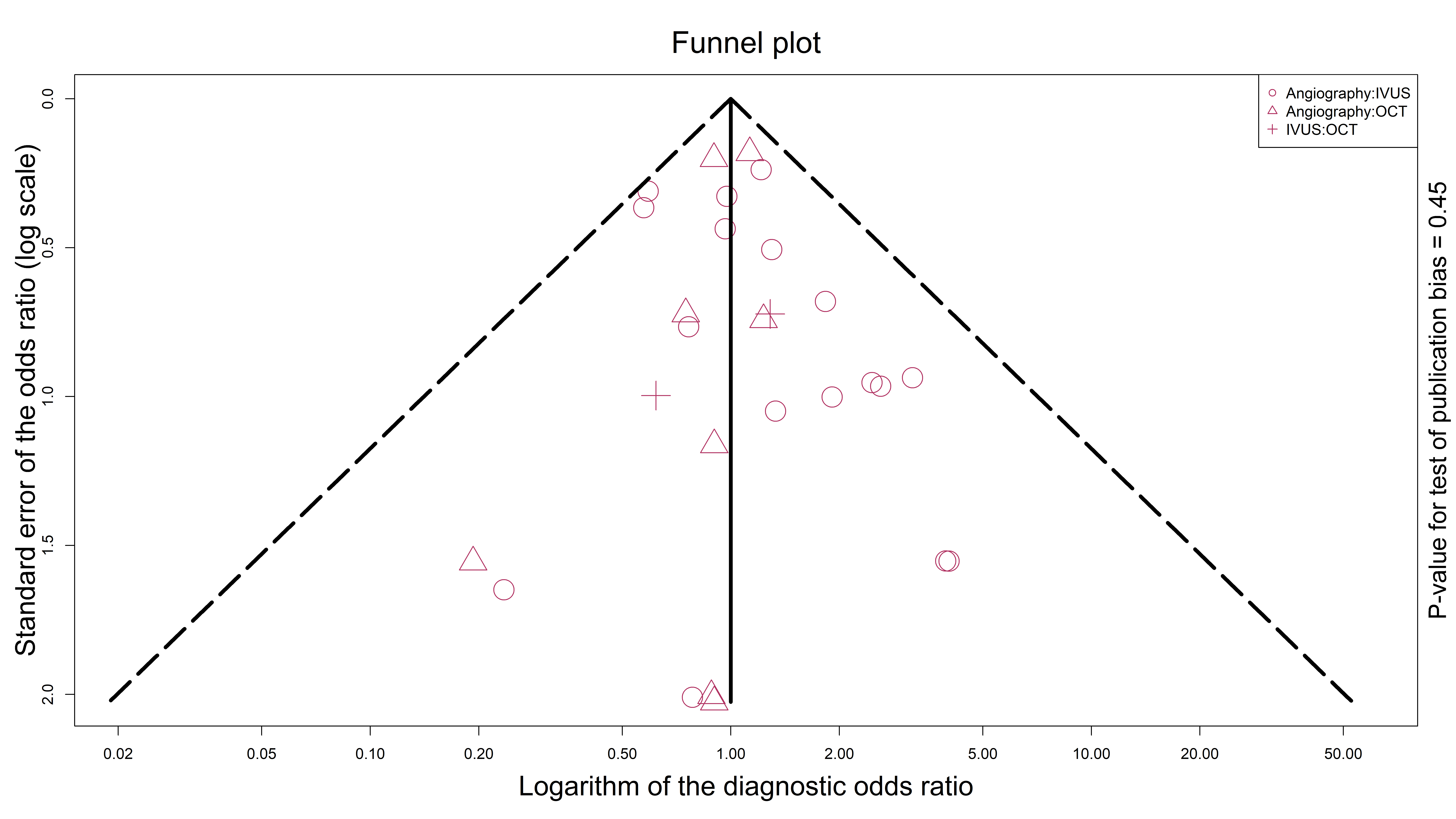
***

***Figure S28: Funnel plot of myocardial infarction.***

***
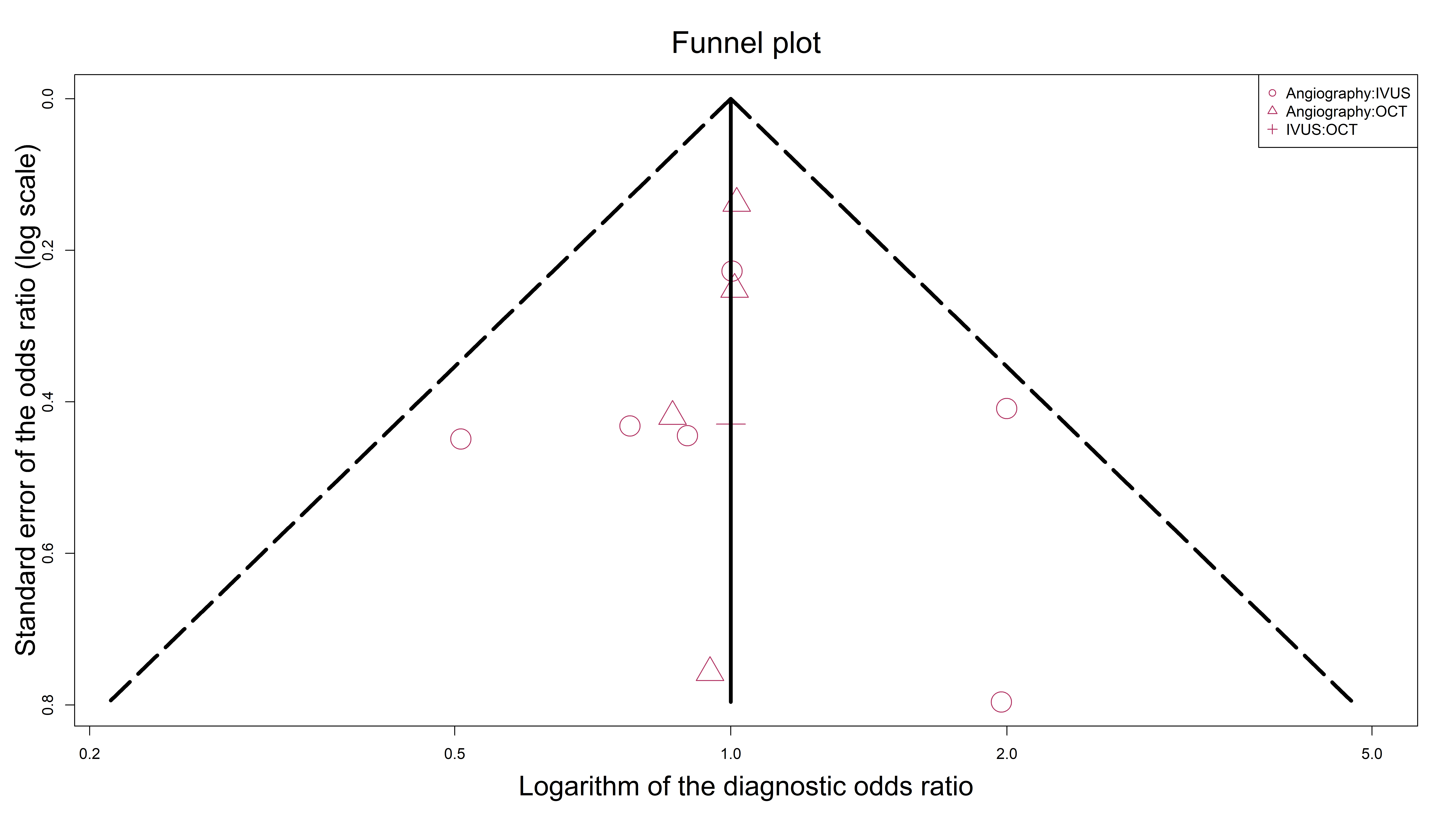
***

***Figure S29: Funnel plot of any revascularization.***

***
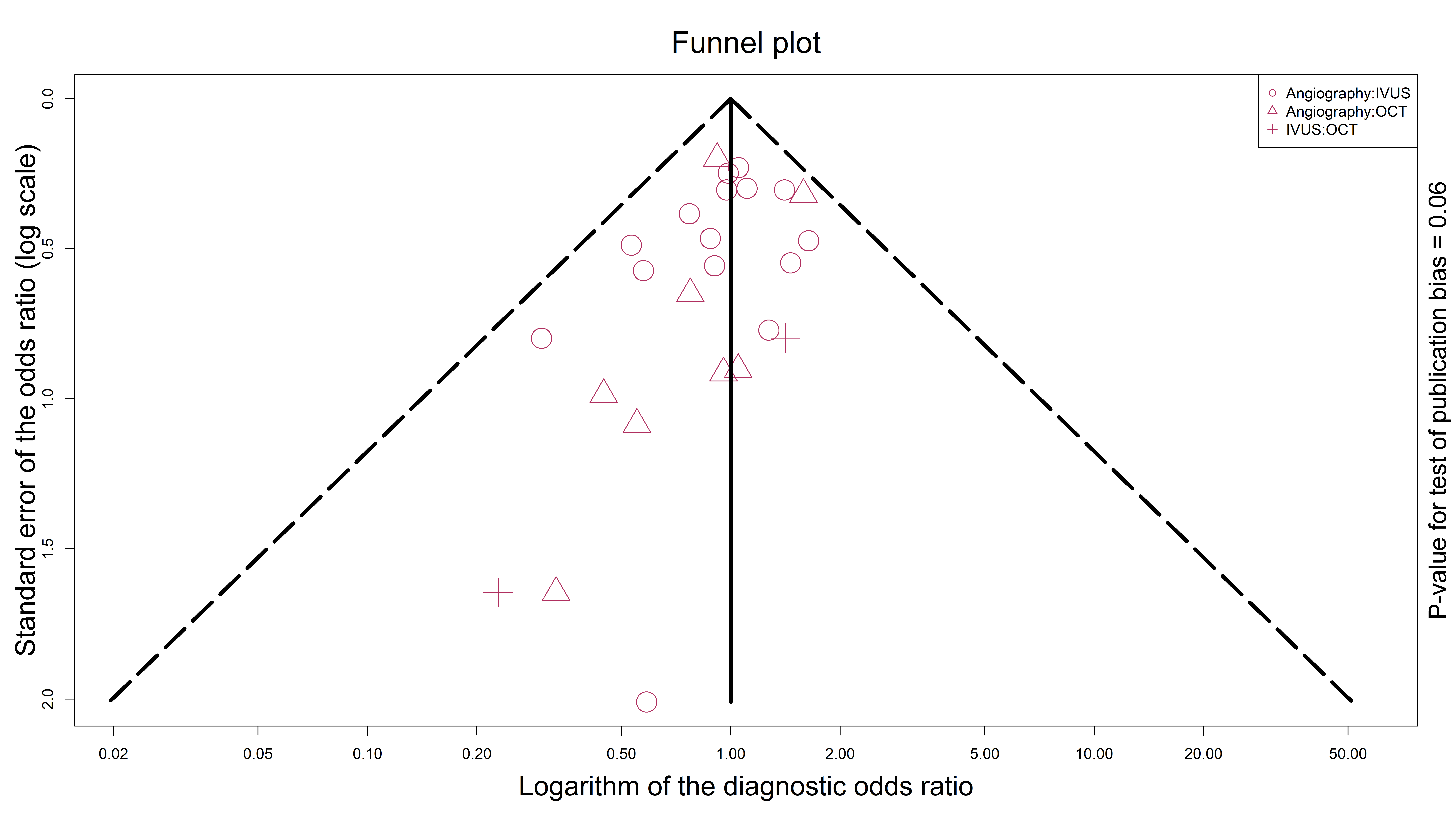
***

***Figure S30: Funnel plot of target-vessel revascularization.***

***
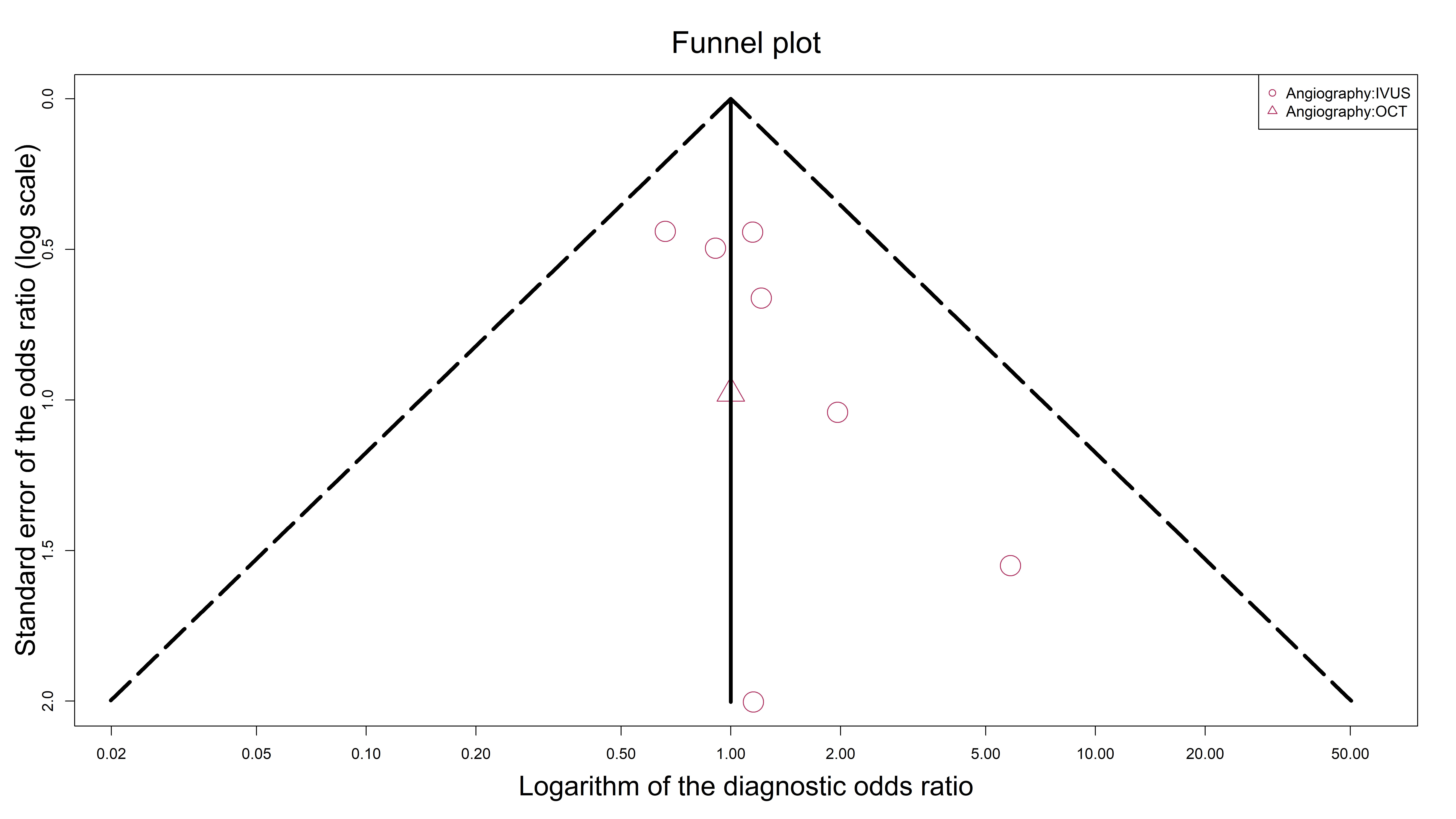
***

***Figure S31: Funnel plot of CABG.***

***
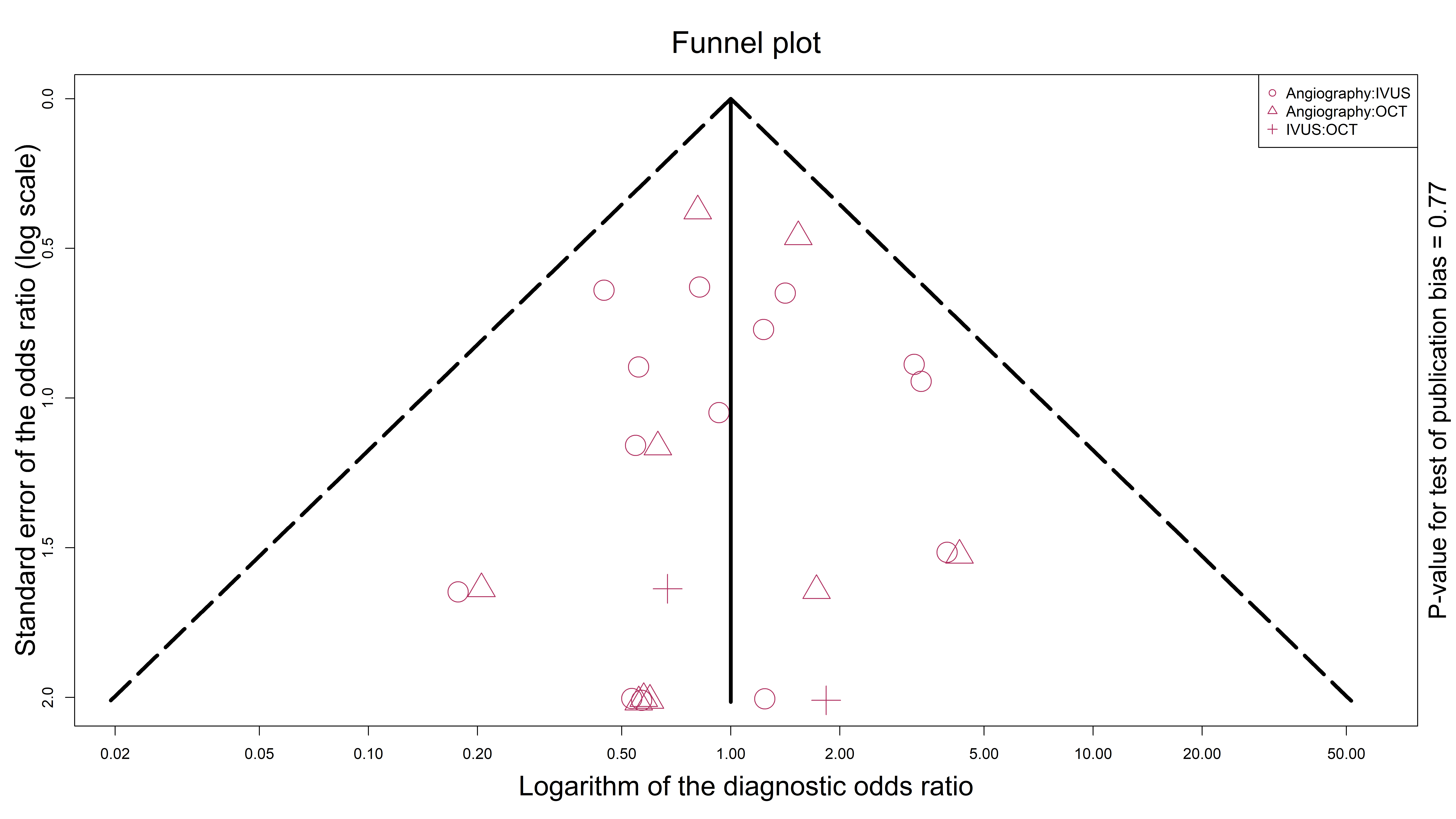
***

***Figure S32: Funnel plot of stent thrombosis.***

***
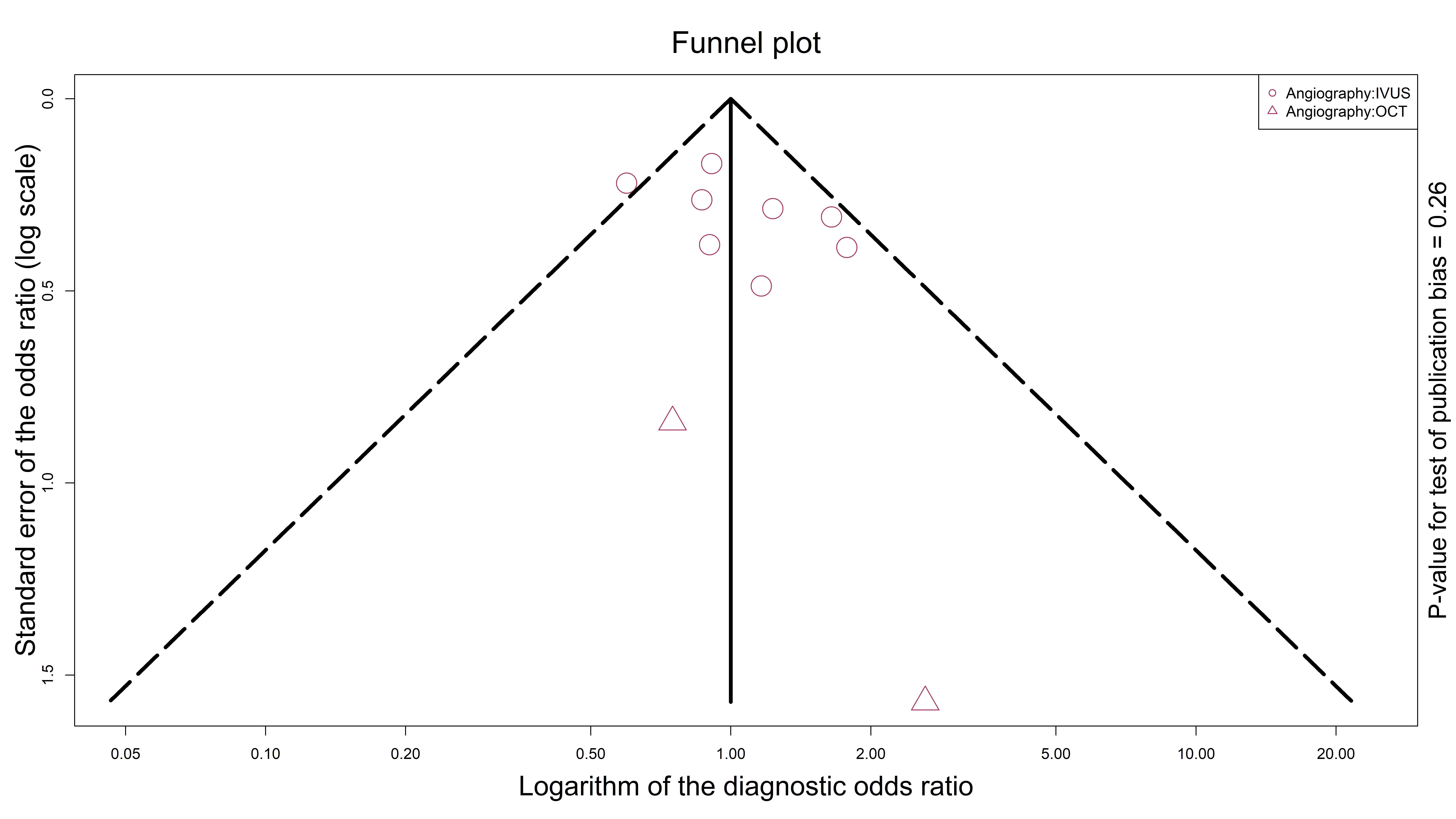
***

***Figure S33: Funnel plot of re-stenosis.***
